# Microbial population dynamics in response to *Pectobacterium atrosepticum* infection in potato tubers

**DOI:** 10.1038/srep11606

**Published:** 2015-06-29

**Authors:** Viia Kõiv, Märt Roosaare, Eve Vedler, Paula Ann Kivistik, Kristel Toppi, David W. Schryer, Maido Remm, Tanel Tenson, Andres Mäe

**Affiliations:** 1 Institute of Molecular and Cell Biology, University of Tartu, 23 Riia Street, Tartu 51010, Estonia; 2 Estonian Genome Center, University of Tartu, Riia 23 B, 51010 Tartu, Estonia; 3 Institute of Technology, University of Tartu, 1 Nooruse Street, Tartu 50411, Estonia

## Abstract

Endophytes are microbes and fungi that live inside plant tissues without damaging the host. Herein we examine the dynamic changes in the endophytic bacterial community in potato (*Solanum tuberosum*) tuber in response to pathogenic infection by *Pectobacterium atrosepticum,* which causes soft rot in numerous economically important crops. We quantified community changes using both cultivation and next-generation sequencing of the 16S rRNA gene and found that, despite observing significant variability in both the mass of macerated tissue and structure of the endophytic community between individual potato tubers, *P. atrosepticum* is always taken over by the endophytes during maceration. 16S rDNA sequencing revealed bacteria from the phyla *Proteobacteria, Actinobacteria, Firmicutes, Bacteroidetes, Fusobacteria, Verrucomicrobia, Acidobacteria,* TM7, and *Deinococcus-Thermus*. Prior to infection, *Propionibacterium acnes* is frequently among the dominant taxa, yet is out competed by relatively few dominant taxa as the infection proceeds. Two days post-infection, the most abundant sequences in macerated potato tissue are *Gammaproteobacteria*. The most dominant genera are *Enterobacter* and *Pseudomonas*. Eight days post-infection, the number of anaerobic pectolytic *Clostridia* increases, probably due to oxygen depletion. These results demonstrate that the pathogenesis is strictly initiated by the pathogen (*sensu stricto*) and proceeds with a major contribution from the endophytic community.

A wide diversity of bacteria interact with plants and form a broad spectrum of communities that often differ between plant species and contribute to the development and health of plants in various ways. Endophytes are often defined as microbes and fungi that live within plant tissues without either damaging the host or eliciting symptoms of plant disease^1^. However, some organisms that have been classified as endophytes seem to be latent pathogens and, under certain conditions, either induce or participate in host infections. Interestingly, both endophytic and pathogenic bacteria utilize similar molecular mechanisms to interact with their plant hosts^2^.

A considerable fraction of endophytes in plant roots originate from soil. It seems that a relatively small number of endophytes are recruited from the large pool of soil species and clones that exist within the bulk soil or the narrow region of soil that interacts with the plant root, termed the rhizosphere. The bacterial community that lives within root tissues, the root endosphere, is less diverse than the communities in the rhizosphere^3^.

In most plants, more endophytes are found in roots than in above-ground tissues^4^. The species composition of the bacterial communities that associate with plants fluctuates depending on the genotype and growth stage of the host plant, environmental conditions, and the type of soil^5^,^6^,^7^,^8^.

Endophytic communities are typically dominated by relatively few bacterial phyla. The most abundant bacterial species found within potato, rice, and *Arabidopsis* tissues are classified under the phyla *Actinobacteria, Bacteroidetes, Proteobacteria,* and *Firmicutes*^5^,^9^,^10^. There is a taxonomically narrow set of core root microbiota from the orders *Actinomycetales, Burkholderiales*, and *Flavobacteriales* found in three *Arabidopsis* species, and these remain stable under both natural and controlled environments^11^,^12^.

In potato (*Solanum tuberosum*), the species composition in the rhizosphere and endosphere depends on both the potato cultivar and the growth stage of the plant^9^,^13^,^14^.

Most plant microbiota studies address growing plants^15^,^16^,^17^,^18^, however, some have examined post-harvest endophyte communities. To the best of our knowledge, the most recent study of the microbial population dynamics within potato tubers during the storage was conducted by Perombelon and coworkers in 1979^19^ and was limited to a few bacterial taxa.

Endophytes have historically been identified using cultivation-based methods. However, not all endophytes can be cultivated due to either unknown growth requirements or the presence of bacteria that are in a viable but not in a cultivatable state^20^. Various molecular techniques provide means to overcome these difficulties^21^,^22^.

We have previously shown that the resident bacterial community within potato tubers can survive in considerable numbers despite surface-sterilization with hypochlorite acid and prior to inoculation with the plant pathogen, *Pectobacterium wasabiae*^23^. Nevertheless, our previous study focused on pathogenesis and did not aim to describe changes within the endophytic bacterial community. Now we aim to cover this gap by describing how the endophytic microbial community within post-harvest potatoes changes in response to soft rot pathogen attack.

## Results

To characterize the microbial communities in potato tuber, we used potatoes harvested in 2012 (Experiment 1) and 2013 (Experiment 2). Experiment 1 was performed using potato tubers that were stored until March 2013 while Experiment 2 was performed using tubers stored until December 2013. Because of this time difference we expected that the results of these experiments would differ while preserving some common features that could be interpreted as reflecting general aspects of changes in the potato microbiome in response to infection with *Pectobacterium atrosepticum* SCRI1043.

**Figure 1:**
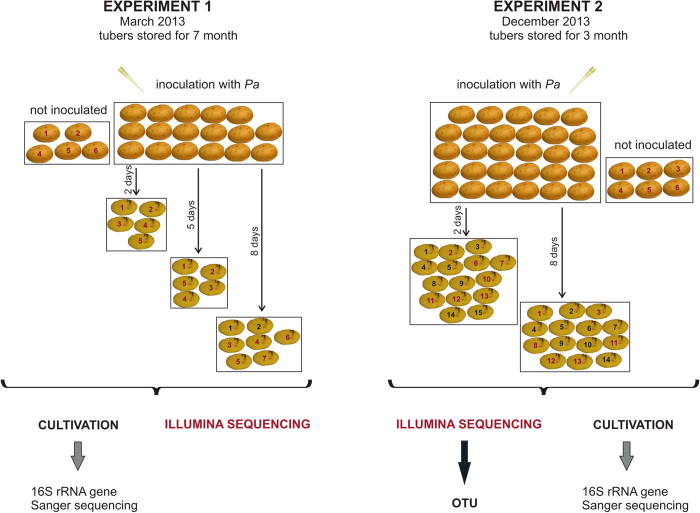
Schematic representation of the experimental setup. Bacteria were cultivated from all the potatoes marked with numbers. Red numbers indicate that the Illumina sequencing was performed in addition to the cultivation. *Pa* designates inoculation with the pathogen, *P. atrosepticum*.

Every potato tuber was infected with the minimum number of *P. atrosepticum* cells (1.5 × 10^4^ cells per ml) required to induce maceration (as determined by preliminary experiments). In Experiment 1, we sampled the macerated tissue from potato tubers incubated for either two, five, or eight days after infection with *P. atrosepticum.* The fifth day of sampling was not performed in Experiment 2 because the bacterial counts determined in Experiment 1 for the fifth and eighth day samples were quite similar. Figure 1 provides an overview of the experimental setup.

### Cultivable bacteria in *P. atrosepticum* macerated potato tissue

**Figure 2:**
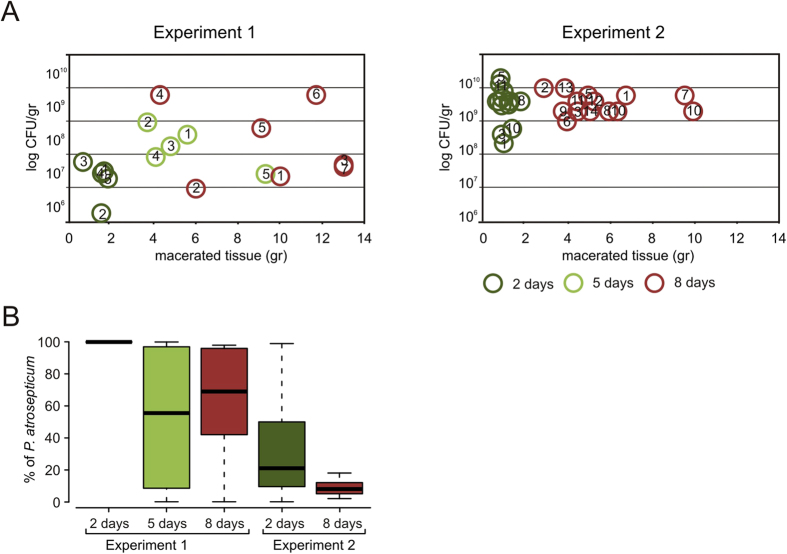
Potato tuber maceration by *P*. *atrosepticum* and resident cultivable microbiome. (**A)** The amount of macerated potato tissue and the content of cultivable bacteria in the macerated tissue. Numbering of potato samples, indicated inside the circle, is recurrent throughout the study. (**B**) The proportion of *P. atrosepticum* in the entire population of cultivated bacteria in the macerated potato tissue.

First, we examined the dynamics of cultivable bacteria in macerated potato tissue. Bacterial CFU counts of macerated potato tuber tissue started to rise quickly (Fig. 2). In both experiments, the CFU/g reached 10^8^–10^10^ by the eighth day post-infection (Fig. 2A). Although the mass of macerated tissue was similar in the two experiments, fluctuations in the CFU/g for individual potatoes were significantly higher in the first. The CFU/g in uninfected potatoes ranged from 2 × 10^4^–10^5^ in both experiments.

The quantity of *P. atrosepticum* among all cultivated bacteria was determined on the basis of 50 randomly picked colonies from each sample. The level of *P. atrosepticum* was quite different in the two experiments, although the mass of macerated tissue was similar (Fig. 2). In Experiment 1, no other bacteria, aside from *P. atrosepticum,* was present among the 50 colonies taken from the macerated tissue two days after infection. Other bacteria appeared on the fifth day post-infection, although the differences between individual potato tubers were remarkable. Similar infection levels occurred by the eighth day post-infection. By the second day after inoculation in Experiment 2, endophytic bacteria had gained ground in the macerated tissue within most tubers. *P. atrosepticum* continued to decrease and did not make up >20% of the total CFU by the eighth day, although the mass of macerated tissue varied between 3 to 11 g per tuber. There was no correlation between the total CFU, % of *P. atrosepticum,* and the amount of macerated tissue in either experimental series (Fig. 2).

**Figure 3:**
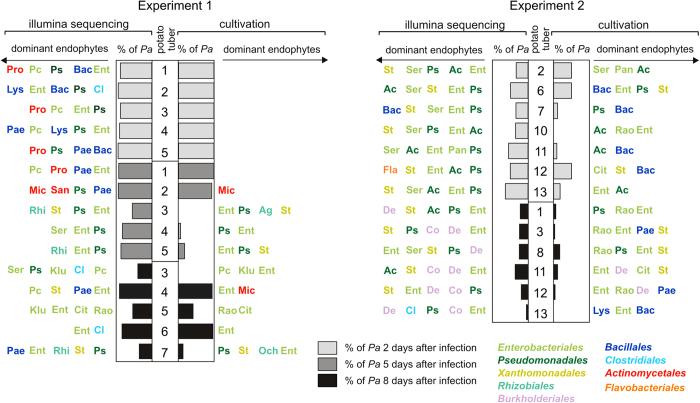
Comparison of cultivation and Illumina sequencing results. The columns indicate the proportion of *P. atrosepticum (Pa) in* the entire population of cultivated and Illumina sequenced bacteria in the macerated potato tissue. Five most prevalent bacterial genera based on cultivation (right-hand side of the columns) and Illumina sequencing (left-hand side of the columns) that are present ≥1% in particular potato sample: Cit - *Citrobacter*; Ent - *Enterobacter*; Klu - *Kluyvera*; Pan - *Pantoea*; Pc - *Pectobacterium carotovorum*; Rao - *Raoultella*; Ser - *Serratia*; Ps - *Pseudomonas*; Ac - *Acinetobacter*; St - *Stenotrophomonas*; Ag - *Agrobacterium*; Och - *Ochrobactrum*; Rhi - *Rhizobium*; Co - *Comamonas*; De - *Delftia*; Bac - *Bacillus*; Lys - *Lysinibacillus*; Pae - *Paenibacillus;* Cl - *Clostridium*; Mi - *Microbacterium*; Pro - *Propionibacterium*; San - *Sanguibacter*; Fla - *Flavobacterium*. Strains are colored according to their respective order. The arrow indicates the direction of decreasing amount of bacteria.

The endophytic bacteria were grouped by phenotypical and morphological characteristics and followed by 16S rDNA sequencing. Altogether, 82 different bacterial strains from four phyla: *Proteobacteria*, *Actinobacteria*, *Bacteroidetes* and *Firmicutes -* were isolated (Fig. 3). The *Gammaproteabacteria* were the most dominant cultivatable taxon and these were largely comprised of bacteria from two groups, *Enterobacteriaceae* and *Enterobacter* (*Lelliottia*). In second place was the *Pseudomonaceae* family with various species of *Pseudomonas*. These two families were those most prevalent in most samples from both experiments.

A higher variability of prevalence in the samples occurred among the bacteria from the phylum *Firmicutes*, and more precisely *Paenibacillus sp.*, *Lysinibacillus fusiformis,* and several *Bacillus* species. The number of *Acinetobacter calcoaceticus* detected was high, however, was exclusively found in the second day post-infection in Experiment 2.

### Amplicon analyses by Illumina sequencing

Five to seven potato tubers were randomly chosen from each time-point in both experiments to follow bacterial community dynamics using rRNA gene mass-sequencing. Note that the designation of potato tubers (Fig. 2) for cultivated bacteria is kept the same for designation of the sequencing results. Approximately 300 bp of the 16S rRNA gene spanning the variable regions V1 and V2 was amplified and sequenced. In Experiment 1, this generated ~100 bp high quality reads that randomly cover the 300 bp V1–V2 region of 16S rRNA gene. In total, 2.6 × 10^6^ reads were obtained with sequences per infected potato ranging from 9 × 10^3^ to 5 × 10^5^ high-quality tags. About 99.9% of the sequence reads obtained from samples of uninfected potato tubers were classified as chloroplasts and not further analyzed. The number of chloroplast reads in the infected potato samples was <1.5%. In Experiment 2, sequencing generated 250 bp high quality reads spanning V1 and V2. In total, 8.5 × 10^7^ reads were obtained with sequences per infected potato ranging from 3.8 × 10^4^ to 8.8 × 10^5^ high-quality tags. As with Experiment 1, ~99.9% reads from uninfected potatoes were classified as chloroplasts and left unanalyzed. As a consequence, the number of reads of bacterial origin from uninfected potato samples was 2–3 magnitudes lower (61–1385 in Experiment 1 and 84–1914 in Experiment 2) than in the samples of macerated potato tissue.

### Survival of *P. atrosepticum* during tuber maceration

**Figure 4:**
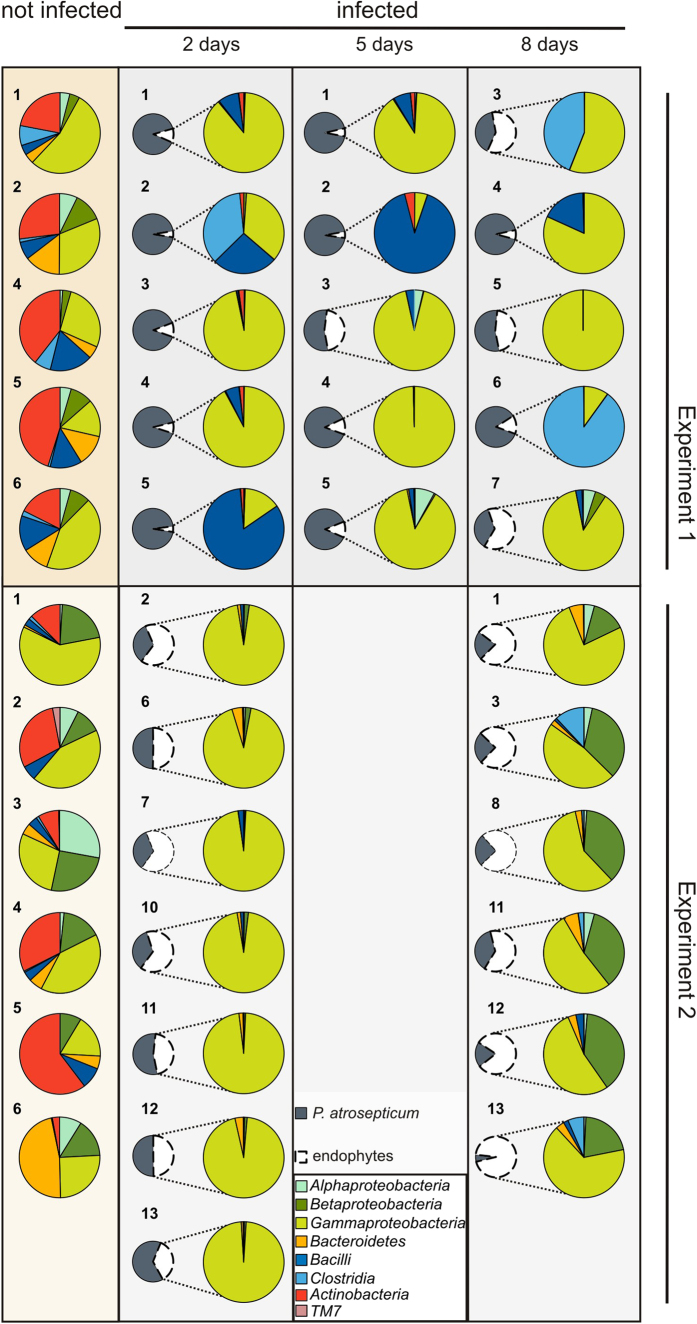
Phyla/class level distribution of all sequence reads from each potato sample. Taxonomic assignments were made using the Ribosomal Database Project (50% confidence threshold). Small pie charts show the percentage of *P. atrosepticum* reads (dark grey 98% identity) among total sequence reads from individual potatoes. The larger pie charts show the composition of the dominant endophytic bacterial community, the colored segments represent bacterial phyla/classes that were most abundant in each sample. (The numbering of potato samples is recurrent throughout the study).

Survival of *P. atrosepticum* was assessed by counting all sequences that had >98% similarity with the *P. atrosepticum* 16S rRNA gene. A 98% cutoff was chosen to exclude reads derived from intrinsic *Pectobacteria* that inhabit the tuber, e.g., the identity of the region V1–V2 of *P. atrosepticum* and *Pectobacterium carotovorum* is 97%. Figure 4 provides the ratio of *P. atrosepticum* reads within the entire dataset of 16S rRNA gene reads. The survival results provided by Illumina sequencing agree with those obtained using bacterial cultivation (Fig. 3). Potatoes sampled in Experiment 1 displayed both higher absolute counts of *P. atrosepticum* and much higher variation in *P. atrosepticum* counts than the potatoes sampled in Experiment 2.

### Diversity of the resident microflora in potato tubers

To assess the structure of the endophytic bacterial community in macerated potato tissue, we excluded *P. atrosepticum* sequences and analyzed the remaining sequences. All high quality reads from both uninfected and infected potatoes were classified using the RDP classifier^24^. Altogether, sequences from phyla *Proteobacteria, Actinobacteria, Firmicutes, Bacteroidetes, Fusobacteria, Verrucomicrobia, Acidobacteria*, TM7, and *Deinococcus-Thermus,* were detected. In both experiments, the dominating sequences all belong to the phyla *Proteobacteria, Firmicutes, Actinobacteria,* and *Bacteroidetes* (Fig. 4).

To characterize the community structures in more detail, sequences from the 19 samples of Experiment 2 were pooled and grouped into operational taxonomic units (OTU) based on >98% identity. The minimal permitted number of reads per OTU was five. Because the 100 bp sequences obtained from Experiment 1 randomly covered the 300 bp V1–V2 region, it was impossible to group these reads to generate OTUs. Therefore, the sequences of Experiment 1 were grouped into the OTUs using the results of Experiment 2 (Supplemental Data 1).

**Figure 5:**
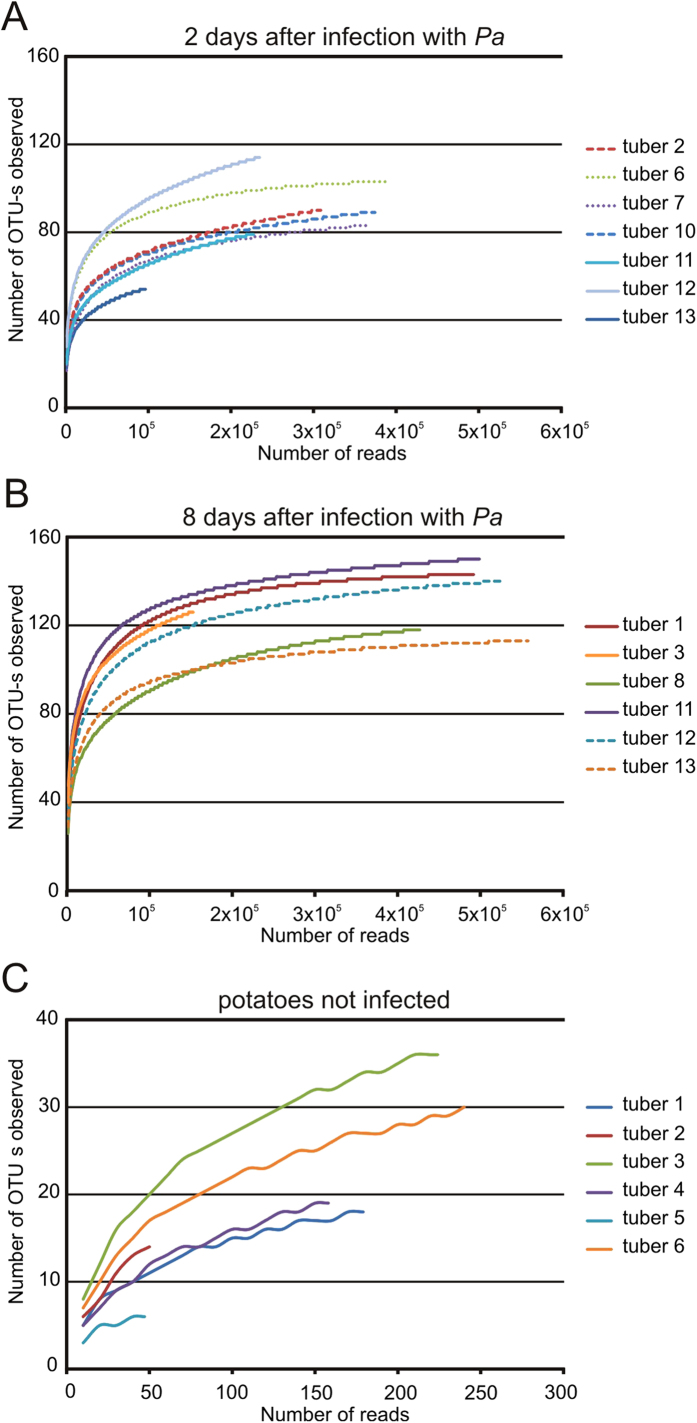
Rarefaction analysis curves of endophytic bacterial 16S rDNA sequences of Experiment 2. Sequences were clustered into OTU - s (≥5 sequences per OTU) using a sequence identity threshold of 98%. (**A**) Macerated potato tuber tissue samples after two days of incubation after infection with *P. atrosepticum (Pa)*; (**B**) Macerated potato tuber tissue samples after eight days of incubation after infection with *P. atrosepticum (Pa)*; (**C**) Uninfected potato tuber samples.

Although the sequencing was very deep (4,780,691 pooled sequences of Experiment 2), only 294 OTUs were detected in Experiment 2. Rarefaction curves show that bacterial diversity, as assessed by the number of OTUs, increased during maceration, starting from 50–114 OTUs on the second day post-infection and reached 113–150 OTUs by the eighth day (Fig. 5). The small amount of OTUs (6–36) detected within uninfected potato samples might be caused by the very low number of sequence reads from these samples, however, most rarefaction curves indicate saturation in the samples (Fig. 5).

**Figure 6:**
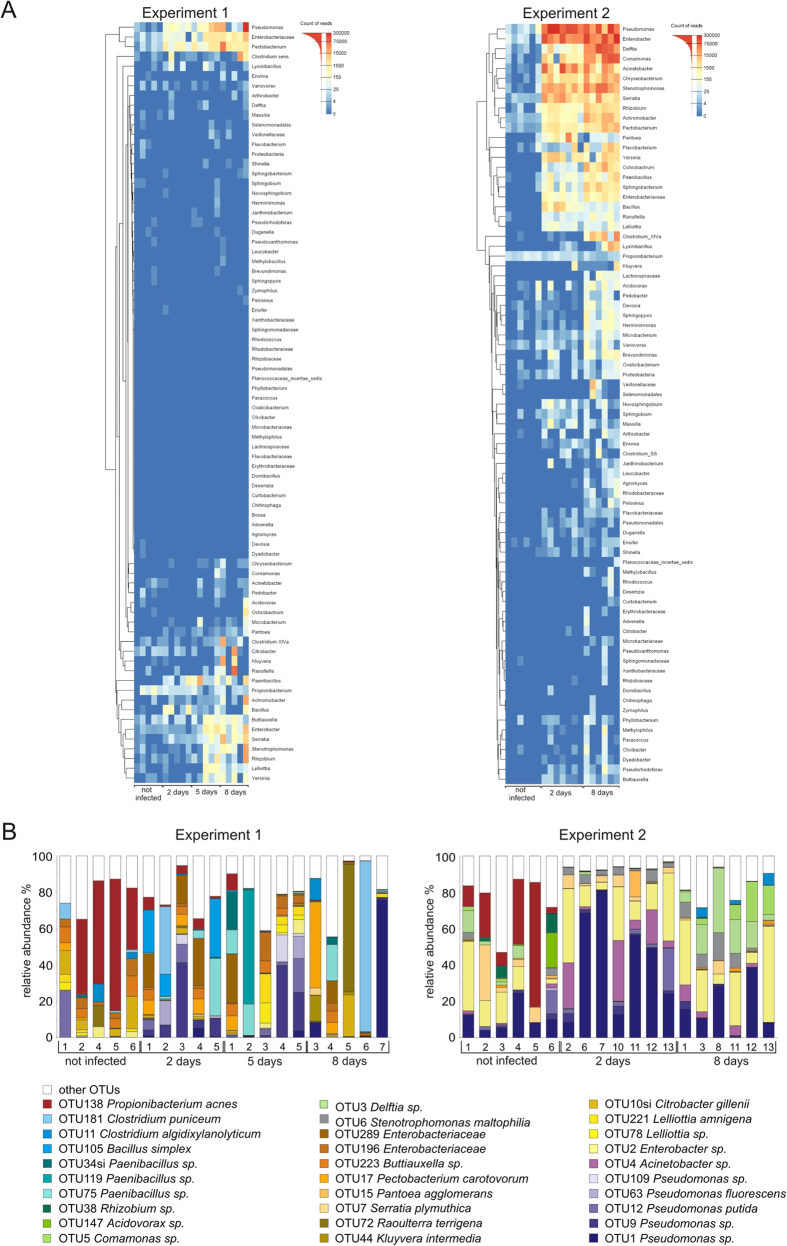
Distribution of endophytic bacteria according to mass-sequencing results. **(A)** Sequencing reads of Experiment 2 were clustered with AbundantOTU; all the chimeric clusters were removed^56^. Consensus sequences (OTUs) were classified using the RDP classifier ^24^ and clustered at the genus level. OTU sequences from Experiment 2 were used to cluster all similar sequences from Experiment 1. Data have been normalized using inverse hyperbolic sine transformation to make a heatmap. Heatmaps were made in R using pheatmap (parameters: clustering_method = average, clustering_distance = euclidean). (**B)** Dominating OTUs that are present ≥10% in any of the samples in Experiments 1 and 2.

OTU sequences were classified at the genus level using the RDP classifier (Fig. 6). In cases where it was impossible to classify the OTUs at the genus level, the next higher taxonomical rank was used. OTUs generated based on the data from Experiment 2 were used to cluster the sequences in Experiment 1, which resulted in over 75% of the sequences being classified. The sequences were counted and used to make a heatmap as in Experiment 2 (Fig. 6A). To see the extent to which OTUs from Experiment 2 represent Experiment 1, the heatmap was based on the entire dataset of Experiment 1 as classified at the genus level by the RDP classifier. Only a few differences between the two heatmaps from Experiment 1 (Fig. S1) were found: the RDP classified total dataset contains more genera with very low sequence counts; a greater abundance of the family *Lachnospiraceae* (samples 3 and 6 from day eight), and of the enterobacterial genera *Yersinia*, *Buttiauxella,* and unclassified *Enterobacteriaceae* (sample 5 from day eight). We conclude that this classification method and analysis is robust.

There were a few very abundant OTUs in the samples from Experiment 2 (Fig. 6B). In the samples from the second day post-infection *Pseudomonas sp*. OTU1, *Lelliottia amnigena,* OTU2 and *Acinetobacter calcoaceticus* OTU4 covered 65–85% of all sequence reads. In the samples from the eighth day, four OTUs dominated, *Pseudomonas sp*. OTU1; *Lelliottia amnigena* OTU2; *Delftia sp* OTU3 and *Comamonas sp.* OTU5, and these covered between 53–80% of all sequence reads. OTU138 (*Propionibacterium acnes*) was highly abundant in uninfected potatoes.

The distribution of OTUs was highly variable in the potato tubers sampled during Experiment 1. For example, *Pseudomonas sp.* OTU1 covered 75% of all sequence reads in the sample 7 of eighth day, but <4% in other infected potato samples, and was not found in uninfected potatoes. The spectrum of dominant bacterial groups proved to be very different from Experiment 2, especially the dominance of *Firmicutes* (*Bacillus, Paenibacillus, Clostridium*). The only clear similarity between the two experiments was the abundance of *Propionibacterium acnes* OTU138 in uninfected potato samples.

### Endophytic population dynamics in *P. atrosepticum* infected potato tubers

The succession of bacteria in *P. atrosepticum* infected potato tuber tissue developed uniformly in Experiment 2. During the first two days of maceration, the fast growing members of family *Enterobacteriaceae* (dominated by *Enterobacter sp*.) and *Pseudomonaceae* (dominated by various *Pseudomonas* strains and *Acinetobacter sp.*) began to flourish in the macerated tissues of all potatoes investigated. During the total progress of maceration (eight days), the *Gammaproteobacteria* were partially taken over by *Betaproteobacteria*, mainly from the closely related genera *Comamonas* and *Delftia* and to a lesser degree by other bacteria who are members of *Alfaproteobacteria, Bacteroidetes, Clostridia,* and *Veillonellaceae*.

In Experiment 1, the endophytic bacterial succession during maceration varied between the different potato tubers sampled: after two days post-infection with *P. atrosepticum* (samples 2 and 5), the members of phylum *Firmicutes - Bacilli* (*Paenibacillus)* and *Clostridia* (*Clostridium puniceum)* - dominate in some potato tubers, but not in others (samples 1, 3, and 4) *Gammaproteobacteria* (*Enterobacter sp*., *Pseudomonas spp*.). The same tendency continued after eight days of infection. In both experiments, the quantity of *Clostridia* increased as maceration progressed, probably as a consequence of oxygen depletion, although the amount remained much lower in Experiment 2 (Fig. 4). The number of representatives of bacteria that start to dominate over *P. atrosepticum* during maceration was already higher before infection, *Betaproteobacteria* in Experiment 2 and *Firmicutes* in Experiment 1 (Fig. 4).

**Figure 7:**
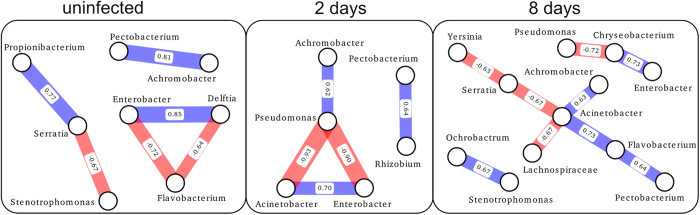
Genera level bacterial correlation networks during *P*. *atrosepticum* infection for Experiment 2. Each network was inferred by analyzing the fractional abundance of bacteria with one set of individual potatoes using SparCC^58^. Connections between nodes give the correlations between genera that form greater than 0.2% of the bacterial population in any given potato within one set. Only connections with correlation values >0.6 and pvalues < = 0.04 are included in the network. Red indicates a negative correlation and blue indicates a positive correlation and the width of each edge is proportional to the pvalue. The most significant relationship is the antagonistic triangle between *Pseudomonas*, *Acinetobacter*, and *Enterobacter* that emerges two days after infection.

It is possible that some taxa have positive or negative interactions between others. In Experiment 1 the complexity is high and considering the low number of potato tubers analyzed any association analysis is of limited value. In Experiment 2 there was a smaller number of dominating taxa and we performed an association analysis^58^. The results indicate that at day two the tubers have either a higher fraction of *Endobacter* (*Lelliottia*) and *Acinetobacter* or *Pseudomonas* (Fig. 7). These associations are not as significant in day eight, however, inspecting the corresponding OTUs presented in Fig. 6B suggests similar trend.

### Comparing bacterial isolates with Illumina sequencing data

Both methods clearly demonstrate that the fraction of *P. atrosepticum* decreases during infection (Fig. 3). As expected, not all of the taxa detected by sequencing are cultivatable under the conditions used. In general, the endophytic bacteria from potato tubers can be efficiently cultivated and the data are in agreement with sequencing results.

**Figure 8:**
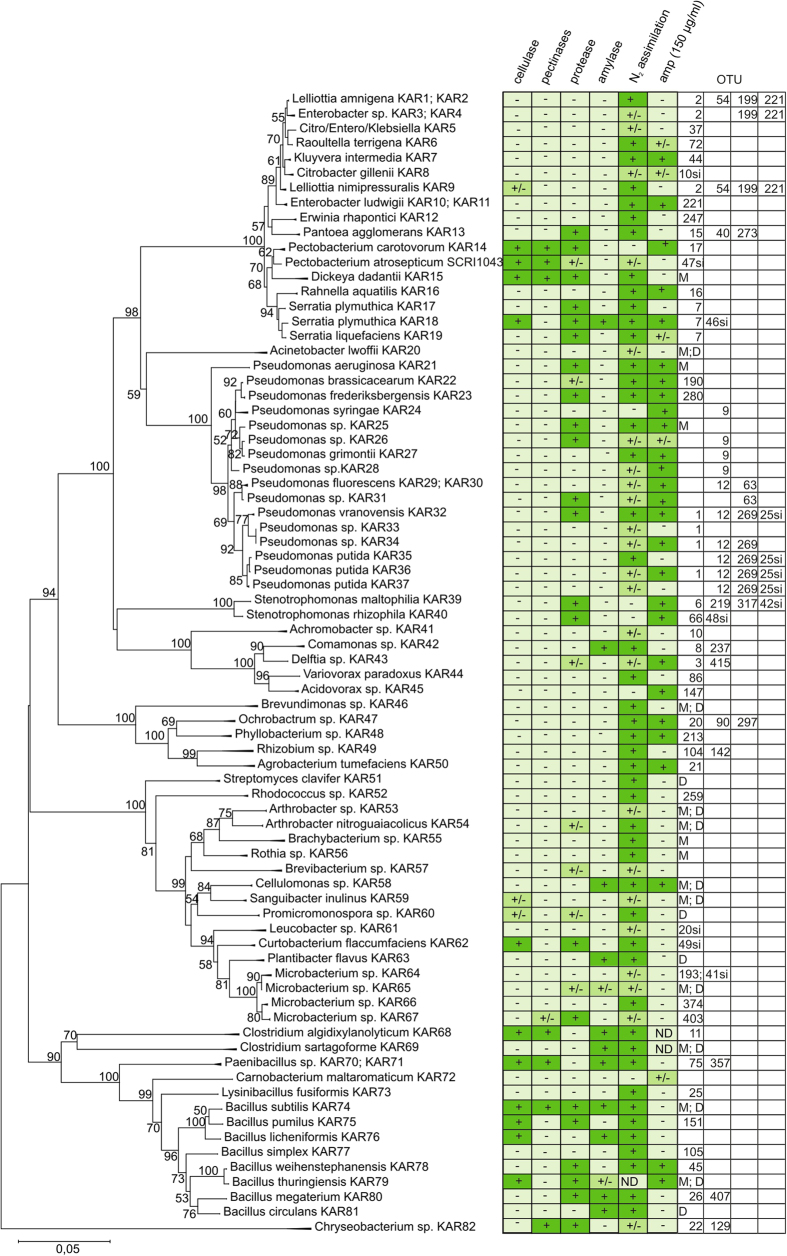
Phylogenetic tree of cultivable bacteria based on the 16S rRNA gene sequence comparison. The tree was constructed from the isolated 16S rRNA bacterial sequences with their respective reference sequences from GenBank. Only bacteria isolated in this experiment are shown as tree branches (full tree is provided in additional Fig. 2). Numbers at the nodes indicate the percentages of occurrence in 1,000 bootstrapped trees; only values >50% are shown. The bar indicates 0.05 substitutions per nucleotide position. The degradation capacity of the main plant polymers CMC - carboxymethylcellulose, PGA-polygalacturonic acid, proteins (casein) and starch are provided. Fixation of N_2_ and sensitivity to ampicillin are also indicated. OTU-s matching to the 16S rDNA of isolated bacteria at >99% identity have been added to the tree. M - sequences in a single sequences pool of Experiment 1 matching with >98% identity to the corresponding cultivated 16S rDNA sequence; D - sequences in single sequences pool of Experiment 2 matching with >98% identity to the corresponding cultivated 16S rDNA.

We investigated how isolated bacteria are represented in the pool of OTUs. The isolates are marked with the letters “KAR” and a corresponding number. To identify OTUs, a BLAST homology search was performed against the 16S rDNA sequences of cultivated strains using >98% sequence identity cutoff. A phylogenetic tree was constructed based on the nearly full-length 16S rRNA gene sequence and their closest evolutionary counterparts retrieved from the GenBank database (Fig. S2). Only bacteria isolated in the present work (KAR) are shown as tree branches in Fig. 8. The phenotypic descriptions and OTUs that display >99% identity to the 16S rDNA of isolated bacteria have also been added to the tree. A 99% identity cutoff was used because the two most abundant bacterial groups in the macerated potato tissue, *Enterobacter (Lelliottia)* and *Pseudomonas* cannot be discriminated with a lower cutoff. *Raoultella terrigena* KAR5 (OTU72), *Kluyvera intermedia* KAR7 (OTU44) and *Citrobacter gillenii* KAR8 (OTU10si) are well differentiated from each other at a 99% identity level, but fall into the same branch as *Enterobacter* isolates.

**Figure 9:**
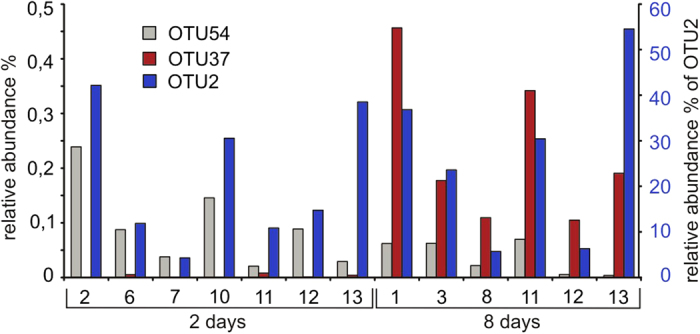
Relative abundance of the main *Enterobacter sp.* OTUs. Individual potatoes are indicated on the x-axis. Values for OTU37 and OTU54 are indicated on the left y-axis and values for OTU2 on the right y-axis.

A disturbance in the 16S rDNA phylogenetic tree is created by isolate KAR5 (OTU37) whose 16S rDNA sequence matches equally well with genera *Citrobacter, Enterobacter,* and *Klebsiella*. After cloning and sequencing 16S rDNA fragments from five clones, we found between one to three nucleotide differences in the sequences of this strain which do not change the phylogenetic affiliation of this strain. The quantity of the strain KAR5 (OTU37) found during the maceration process was distinctive; it increases during maceration while other *Enterobacter* strains decrease (Fig. 9).

As with the *Enterobacter* group, the most abundant *Pseudomonas* strains that belong to the *Ps. putida* group (OUT1; OTU12; OTU269, and OTU25si) and the *Ps. fluorescence* group (OTU9, OUT12 and OTU63) cannot be discriminated based on their 16S rDNA sequences, while other less abundant strains are clearly distinctive (Fig. 8).

Most of the isolates are represented by Illumina sequencing reads with minor exceptions: *Brevibacterium sp*. KAR46, *Carnobacterium maltoaromaticum* KAR72 and *Bacillus licheniformis* KAR76. The number of isolates that are represented with <5 16S rDNA sequences, i.e. not used to generate an OTU, are quite high. The absence of sequences that belong to *Bacillus licheniformis* is unexpected because this easily cultivatable and recognizable bacterium was present in a number of potato samples (data not shown). The proportion of *Bacilli* in rDNA sequencing samples was also clearly lower compared to cultivated samples (Fig. 3).

Reversing the analyses to examine how well the sequence reads are covered by cultivatable taxa showed that most of the dominant bacteria can be cultivated, including *Clostridia* which was isolated anaerobically from frozen samples after its presence was detected by mass sequencing. The anticipated number of different OTUs (294) proved to be more numerous than the number of cultivated bacterial strains (82). One dominant bacterial strain from the macerated tissue from Experiment 2, *Acinetobacter calcoaceticus* (OTU4), was not found in our library of cultivated bacteria which we isolated and identified by MALDI phenotyping, but lost during the purification process. This also occurred with some less dominant *Actinobacteria spp*.

### Phenotypic characterization of isolated bacterial strains

Microbial endophytes that colonize potato tissue are widespread and some of the non-pathogenic endophytes could turn into phytopathogens that are able to induce infection symptoms. The virulence potential of potato endophytes was assessed by their ability to produce extracellular enzymes and their ability to attack plant cell wall components (the most important virulence factors of soft rot bacteria). Degradation of pectic substances (PGA), cellulose (CMC), starch and proteins (casein), was tested on indicator plates. Bacteria that belong to the phylum *Firmicute*s, *Paenibacillus sp, Bacillus subtilis,* and the anaerobic species *Clostridium algidixylanolyticum,* can all degrade the main polymers in a potato tuber, including pectin, cellulose, and starch (Fig. 8). The Enterobacterial plant pathogens, *Pectobacterium carotovorum* and *Dickeya dadantii,* degrade plant cell wall components, but not starch. Some *Bacilli,* *Chryseobacterium sp., Curtobacterium flaccumfaciens,* and *Sanguibacter inulinus,* can hydrolase either pectin or cellulose. Starch is degraded by only a few bacteria and their number tended to increase during the maceration process when the slow growing and oxygen-sensitive *Clostridia* begin to dominate over other bacteria.

To characterize potential symbionts, bacterial assimilation of N_2_ under conditions of low oxygen was followed by the growth of bacteria on nitrogen deficient agar medium modified from a previously published method^25^. This modified medium allows one to grow a wider spectrum of bacterial taxa than those variants previously reported. We found that N_2_ assimilation is intrinsic to the majority of endophytic bacteria in potato. This feature was less common among pseudomonads and representatives of the phylum *Actinobacteria*, however, we might not have found a suitable medium to detect N_2_ assimilation for these bacteria.

While isolating pseudomonads on LB agar supplemented with ampicillin to eliminate other endophytic ampicillin-sensitive bacteria, some *Pseudomonas* strains also seemed to be sensitive to ampicillin. Ampicillin sensitivity of all isolated bacteria was therefore explored. Unexpectedly, the sensitivity of bacteria was not predictable by their systematic affiliation. Several enterobacteria and bacilli are not sensitive, while pseudomonads can be sensitive to ampicillin at 150 μg/ml, a technique generally used to distinguish these bacteria.

## Discussion

The recent development of second generation sequencing techniques has enabled considerable advances in describing plant endophyte communities; however, much less attention has been given to study changes in the composition of the microbial community during plant decay. Here, we infected surface-sterilized potatoes with the pectolytic plant pathogen *P. atrosepticum* using a method adopted in the laboratory to study plant-pathogen interactions. The experiment was carried out twice with two main differences between them, (i) the year of harvesting (and thereby conditions of plant cultivation) and (ii) the length of storage. Although the results of the two experiments are variable, several common features were observed.

1. Sequencing analysis shows the presence of bacteria in tubers from the phyla *Proteobacteria, Actinobacteria, Firmicutes, Bacteroidetes, Fusobacteria, Verrucomicrobia, Acidobacteria,* TM7, and *Deinococcus-Thermus*. This agrees with other reports that have demonstrated that the members of phyla *Proteobacteria, Actinobacteria, Firmicutes,* and *Bacteroidetes* dominate in several plants, including potato^9^,^26^,^27^. The most abundant species in macerated potato tissues belong to the class, *Gammaproteobacteria* - *Pseudomonas sp.* (*putida*) and *Enterobacter sp. (Leliottia).* Strains of the genus *Enterobacter* frequently are described in studies on different plant endophytes^8^,^28^,^29^,^30^,^31^. These strains are commensals, or non-pathogenic microbes, that invade the intercellular space of the host, but are usually dormant. They often have a close relationship with the host plants, as suggested by their widespread ability to assimilate N_2_. We found that, despite their mutualistic nature, these strains begin to proliferate exponentially when pectolytic pathogens initiate an infection process. The same is true for pseudomonads, which are well known plant growth-promoting bacteria^8^,^29^,^32^,^33^, yet begin to flourish in *Pa-*infected potato tubers. *Pseudomonas* and *Enterobacter* strains probably do not help *P. atrosepticum* to decay tuber tissue directly, but utilize sugars and sugar acids liberated during the decay process. Our data also suggest that the pathogenesis process can proceed with the domination of either *Pseudomonas* or *Enterobacter*. This interaction might be considered when analyzing other datasets on plant microbiomes.
2. Like many genera within the family *Enterobacteriaceae*, *Enterobacter* is polyphyletic based on the 16S rRNA gene^34^,^35^,^36^. A possible reclassification of *E. amnigenus* and *E. nimipressuralis* into the novel genus *Lelliottia* as *Lelliottia amnigena* comb. nov. and *Lelliottia nimipressuralis* comb. nov., respectively, was introduced^36^. The dominant enterobacterial isolates in our infected potato tubers belong to this genus, but cannot be discriminated at the species level by their 16S rDNA sequences. The high number of diverse *Enterobacter/Lelliottia* strains in the macerated potato tissue can form a platform for specialization into diverse micro niches during infection, thereby increasing the fitness of this taxonomic group.
3. In both experiments, bacteria that are rare at the beginning of the infection begin to multiply during maceration. In Experiment 2, *Comamonas*, *Delftia,* and other closely related betaproteobacteria, started to out-compete the sugar-consuming *Enterobacter* and *Pseudomonas* strains. Bacteria that belong to the family *Comamonadaceae* prefer organic acids and amino acids rather than carbohydrates, and can degrade a wide variety of complex aromatic compounds (according to Bergey’s Manual of Systematic Bacteriology). The *Comamonas sp*. KAR42 that we isolated also possess amylase activity that may provide a growth advantage in potato tubers. Another large group of bacteria that can dominate over gammaproteobacteria during the maceration process is comprised of different members from the phylum *Firmicutes* (mainly in Experiment 1). These are the most versatile decomposers of plant polymers, namely *Paenibacillus sp.* KAR70/KAR71, *Bacillus subtilis* KAR74 and *Clostridium algidixylanolyticum* KAR68, which can all degrade the main polymers found in potato tubers - pectin, cellulose, and starch. With respect to plant pathogenicity, the possession of pectolytic and to some extent cellulolytic activity is of greater importance to bacteria than amylolytic activity. This expected distribution of enzymatic activities was indeed observed in the endophytes of potato tubers (Fig. 8). The number of anaerobic *Clostridia* begins to increase during maceration, and in some potatoes, *Clostridia, Clostridium puniceum* (OTU181) and *Clostridium algidixylanolyticum* KAR68 (OTU11), overgrew both *P. atrosepticum* and other bacteria. *Clostridium* is one of the major cellulolytic/pectinolytic and fermentative bacterial groups in anaerobic environments, however, few reports discuss bacteria that are able to degrade pectic substances within the genus *Clostridium*. Between 1970–1980, it was found that pectolytic, *i.e*. pathogenic *Clostridia,* can frequently be isolated from decaying plant material. *Clostridia* with high pectolytic and tuber-rotting capabilities were described both in the United Kingdom^37^ and the United States of America^38^. *Clostridium puniceum* may cause potato soft rot^39^ and pectolytic *Clostridia* causes cavity spots in carrots^40^. *Clostridium puniceum* and *C. saccharobutylicumstrain* P262 have been found to cause post-harvest disease in sweetpotato^41^. We observed that *Paenibacillus sp., Clostridium algidixylanolyticum,* and *Clostridium puniceum*, not only support *P. atrosepticum* during plant decay, but in some cases dominate *P. atrosepticum* within the infection site. This is probably because these organisms possess amylase activity^29^ and while *P. atrosepticum* cannot degrade starch directly, it does provide access to an underutilized nutrient niche. Overall, the succession of bacteria in *P. atrosepticum* infected potato tuber could proceed as follows: *P. atrosepticum* breaks down the plant cell wall thereby generating large amounts of free sugars and sugar acids. Bacteria (*Enterobacteriaceae* and *Pseudomonadaceae*) that consume these energetically favorable sugars have an advantage during this phase of infection. Later, the bacteria that specialize in consuming energetically less favorable compounds (*Comamonadaceae*) begin to take over the infection site. During tuber tissue consumption, oxygen is depleted and the diffusion of atmospheric oxygen into the macerated tissue is reduced because most of the bacteria produce slime during this growth phase. Thus, strictly anaerobic *Clostridia,* which can degrade cell wall components, begin to expand and thereby extend tuber decay together with *P. atrosepticum,* or even supersede it.
4. N_2_ fixation is widespread among bacterial plant endophytes. Diazotrophy is well described among *Rhizobiaceae*, but also among plant-associated *Enterobacteriaceae*, *Bacilli* and other species^42^,^43^,^44^. Relying on genome data, the chromosome of plant pathogen *P. atrosepticum* strain SCRI1043 that was used in our potato infection experiments contains genes for molybdenum-dependent nitrogenase synthesis, an enzyme that catalyzes the fixation of nitrogen. However, the ability of *P. atrosepticum* to fix N_2_ was found to be very weak under our experimental conditions. Another enterobacterial plant pathogen, *Pectobacterium carotovorum* KAR14, did not fix N_2_ under these experimental conditions, but *Dickeya dadantii* KAR15 did so quite efficiently (Fig. 8). *Bacillus subtilis* KAR74, *Paenibacillus sp.* KAR70/71 and *Clostridium algidixylanolyticum* KAR68, which can efficiently degrade all plant polymers, are also efficient in N_2_ fixation. Consequently, bacteria that behave as pathogens during some stages of their life can also be mutualistic (i.e. beneficial to the host) at other stages^45^.

An interesting finding is the abundance of actinobacterial *Propionibacterium acnes* in uninfected potato samples, which might have been caused by human contamination when handling the tubers during harvest time and storage. Another explanation is based on recent finding that the human opportunistic pathogen, *Propionibacterium acnes,* could have been horizontally transferred to the grapevine, *Vitis vinifera,* possibly during the Neolithic period when the grapevine was domesticated^46^. We speculate that this phenomenon could be more general, and *Propionibacterium acnes* may have extended the host circle to include domesticated potato.

In conclusion, our data show that bacterial plant pathogenesis is not a process where the role of a particular pathogen can be singled out but involves complex contributions from the endophytic community.

## Materials and Methods

### Plant material and sampling

The commercial potato (*Solanum tuberosum*) cultivar “Ants” was grown under normal field conditions at the Jõgeva Plant Breeding Institute in two consecutive years (2012 and 2013). The soil characteristics of the experimental fields and crop protection measures were similar in both experiments (Supplemental material). The tubers were stored at 7 °C and 92% humidity post-harvest prior to randomly sampling healthy tubers with equal size within the weight range of 80–90 grams. Potatoes harvested in 2012 were stored until March 2013 and used to conduct Experiment 1, while those harvested in 2013 were stored until December 2013 and used to conduct Experiment 2.

### Surface sterilization and virulence assays

Potato tubers were first washed with tap water followed by surface sterilization in 10% sodium hypochlorite (NaOCl) for 10 min, followed by three rinsing cycles with sterile water and an additional rinse with 96% ethanol prior to air-drying. Each tuber was placed into a plastic seedling box on top of a moist sterile paper towel. *Pectobacterium atrosepticum* SCRI1043 (herein referred to as *Pa*)^47^ was grown overnight in M9 medium^48^ supplemented with 0.4% polygalacturonic acid (PGA) as the sole carbon source. The cells were spun down at 5000 g for 5 min. The supernatant was passed through a filter with pore size 0.2 μm. The cells were washed once with 10 mM MgSO_4_ prior to centrifugation and then suspended in the sterile supernatant from the overnight culture. The tubers were stabbed with a pipette tip to create a cavity to inoculate 30 μl of diluted bacterial suspension (5 × 10^5^ cells per ml). The tubers were then incubated in the dark at room temperature and 100% humidity for either two, five, or eight days. At the end of the incubation period, each potato was cut in half and the softened tissue was scraped out, weighed, and used to identify and quantify the composition of its bacterial community. The macerated tissue was frozen and stored at −80 °C prior to DNA extraction.

For the isolation of endophytic bacteria from uninfected potatoes, 15 g from each surface-sterilized tuber, including peel, were cut into pieces and homogenized with a garlic smasher. About 15 ml sterile 0.9% NaCl was added to the mixture, vortexed vigorously, and filtered through cotton fabric (instruments and materials for this procedure were sterile). The filtrate was centrifuged for 30 min at 5000 g and the resulting pellet was used for the extraction of genomic DNA.

### Counting, isolation and identification of cultivable bacteria

The macerated and crude uninfected potato tissue was homogenized together with an equal volume of sterile 0.9% NaCl. Dilutions for CFU counting were plated on R2A agar (LabM), and the plates were incubated for one week at room temperature. We then determined the CFU/g of either macerated or crude tissue for each potato tuber. To distinguish between different bacterial strains, including *P. atrosepticum,* 50 colonies were successively picked from R2A agar and streaked on six different agar plates containing: carboxymethylcellulose (CMC), polygalacturonic acid (PGA), skimmed milk for semi-quantitative agar plate assays for the production of extracellular cellulases; hydrolyses of pectic substances; and proteases^49^. After 2–5 days of incubation, the exoenzyme activity was measured according to the halo on the agar plates, where the size is proportional to the amount of secreted enzyme. Agar plates containing 0.2% starch were used for the detection of amylase activity^50^. The presence of clearance halo after Lugol staining was observed after 5–10 days of incubation. Luria–Bertani (LB) agar medium was supplemented with ampicillin (150 μg/ml) or polymyxin B (5 μg/ml)^51^. Assimilation of N_2_ was studied on the agar plates containing (gL^−1^): MgSO_4_ 0.2; K_2_HPO_4_ 0.8; KH_2_PO_4_ 0.2; CaSO_4_ 0.13; FeCl_3_ 0.00145; Na_2_MoO_4_ 0.000253; sucrose 10; mannitol 10; agar 15 (modified protocol of Wang *et al.*^25^). The plates were kept under microaerophilic conditions for between one to two weeks. All incubations were performed at room temperature.

Bacteria were grouped on the basis of their enzymatic activities and morphological structure. In Experiment 1, representatives of the groups were identified by 16S rRNA gene sequencing. In Experiment 2, the first round of identification was made by MALDI-TOF phenotyping (Bruker Daltonik MALDI Biotyper), which was later confirmed by 16S rRNA gene sequencing. All isolates were identified by Sanger sequencing ~1500 bases of a near complete section of the 16S rRNA gene, using the primer pair PCRI 5′AGAGTTTGATCATGGCTCAG and PCRII 5′TACGGTTACCTTGTTACGACTT adapted from the primers fD2 and rP1^52^. Phylogenetic affiliations of the sequences obtained from this method were assessed using the Ribosome Database Project Classifier^24^. The 16S rRNA gene nucleotide sequences of isolated bacterial strains were deposited in the GenBank database under accession numbers KAR1 - KR054963; KAR2 - KR054964; KAR3 - KR054965; KAR4 - KR054966; KAR5 - KR054967; KAR6 - KR054968; KAR7 - KR054969; KAR8 - KR054970; KAR9 - KR054971; KAR10 - KR054972; KAR11 - KR054973; KAR12 - KR054974; KAR13 - KR054975; KAR14 - KR054976; KAR15 - KR054977; KAR16 - KR054978; KAR17 - KR054979; KAR18 - KR054980; KAR19 - KR054981; KAR20 - KR054982; KAR21 - KR054983; KAR22 - KR054984; KAR23 - KR054985; KAR24 - KR054986; KAR25 - KR054987; KAR26 - KR054988; KAR27 - KR054989; KAR28 - KR054990; KAR29 - KR054991; KAR30 - KR054992; KAR31 - KR054993; KAR32 - KR054994; KAR33 - KR054995; KAR34 - KR054996; KAR35 - KR054997; KAR36 - KR054998; KAR37 - KR054999; KAR39 - KR055000; KAR40 - KR055001; KAR41 - KR055002; KAR42 - KR055003; KAR43 - KR055004; KAR44 - KR055005; KAR45 - KR055006; KAR46 - KR055007; KAR47 - KR055008; KAR48 - KR055009; KAR49 - KR055010; KAR50 - KR055011; KAR51 - KR055012; KAR52 - KR055013; KAR53 - KR055014; KAR54 - KR055015; KAR55 - KR055016; KAR56 - KR055017; KAR57 - KR055018; KAR58 - KR055019; KAR59 - KR055020; KAR60 - KR055021; KAR61 - KR055022; KAR62 - KR055023; KAR63 - KR055024; KAR64 - KR055025; KAR65 - KR055026; KAR67 - KR055027; KAR68 - KR055028; KAR69 - KR055029; KAR70 - KR055030; KAR71 - KR055031; KAR72 - KR055032; KAR73 - KR055033; KAR74 - KR055034; KAR75 - KR055035; KAR76 - KR055036; KAR77 - KR055037; KAR78 - KR055038; KAR79 - KR055039; KAR80 - KR055040; KAR81 - KR055041; KAR82 - KR055042.

The 16S rDNA sequences of isolates (marked as “KAR”) were combined with the closest matching sequences from the Ribosome Database Project^38^. A phylogenetic tree (additional Fig. 2) was constructed in MEGA6^53^ using the neighbor-joining algorithm with “maximum composite likelihood” as the substitution model. Bootstrapping with 1000 repetitions was used to test the correctness of the tree. A smaller tree (Fig. 8) was derived by condensing the reference sequences with those of the isolates. The resulting branches were named after the isolated “KAR” sequences and all branches that had <50% bootstrap support were removed.

### Isolation and cultivation of *Clostridia*

Macerated potato tuber tissue was incubated in 50% ethanol for 1h and streaked on Fastidious Anaerobic Agar plate (FAA) and 10% potato extract containing agar plates, which were kept under anaerobic conditions for one week either at 37 °C or room temperature.

### Total-community DNA isolation

DNA was isolated from 250 mg of bacterial cells obtained from either macerated or crude potato tuber tissue. The cells were suspended in the lysis buffer with beads from a PowerSoil DNA kit (MoBio, USA). The cells were lysed by bead beating with Precellys® 24 (Bertin Technologies, France) at 5000 rpm for 3 × 60 s followed by purification of the extract with phenol, phenol:chloroform:isoamylalcohol (25:24:1) and chloroform:isoamylalcohol (24:1), and subsequently isopropanol precipitation. The quantity and quality of DNA extracts were determined spectrophotometrically (NanoDrop 2000c).

### Amplification of the bacterial 16S rRNA genes

The taxonomic composition of the endophytic bacterial community was also analyzed using deep-sequencing by amplifying the hypervariable V1–V2 region of the 16S rRNA gene using an 8R/357R primer set^54^. PCR amplifications were performed in triplicate and then pooled. The PCR reaction was carried out at 95 °C for 5 min followed by 15 cycles each of 95 °C for 45 s, 50 °C for 45 s, and 72 °C for 30 s, followed by 10 min at 72 °C. The pooled PCR products were cleaned using an UltraClean PCR Clean-Up Kit (MoBio, USA), and both the quantity and quality was determined spectrophotometrically (NanoDrop 2000c). Sequencing used an Illumina MiSeq (San Diego, USA).

### Sequencing of 16S rDNA

In Experiment 1 (March 2013), 20 bacterial 16S rRNA gene amplicons were prepared for sequencing using Nextera XT sample preparation kits (Illumina, San Diego, USA). The purified DNA of the amplicons was first quantified with a Qubit 2.0 Fluorometer (Invitrogen, Grand Island, USA) and 2200 TapeStation (Agilent Technologies, Santa Clara, USA). A sample containing 1 ng of DNA was used to prepare the sequencing libraries using the NexteraXT protocol. Libraries were validated by qPCR with Kapa Library Quantification Kit (KapaBiosystems, Woburn, USA) to optimize cluster generation. Twenty ssDNA Nextera XT libraries were pooled and sequenced on a MiSeq v2 flowcell with paired-end (PE), 250-bp reads yielding in 20 M reads (Illumina CSPro lab, Estonian Genome Centre, University of Tartu, Estonia).

In Experiment 2 (December 2013), 19 bacterial 16S rRNA gene libraries were constructed by two-step PCR. Two 5′overhang Illumina adapters,

Forward primer 5′TCGTCGGCAGCGTCAGATGTGTATAAGAGACAG and

Reverse primer 5′GTCTCGTGGGCTCGGAGATGTGTATAAGAGACAG,

were added to the 8F /357R primer set and used in the first round of PCR, with purification carried out as described above. Purified amplicons were quantified using Qubit 2.0 Fluorometer (Invitrogen, Grand Island, USA) and 2200 TapeStation (Agilent Technologies, Santa Clara, USA). A sample containing 1 ng of DNA was used in the second round of PCR (12 cycles) with the primers from the Nextera XT sample preparation kit. PCR products were purified and normalized using the Nextera XT protocol provided by Illumina. Libraries were validated by qPCR with Kapa Library Quantification Kits (Kapa Biosystems, Woburn, USA) to optimize cluster generation. In total, 19 ssDNA libraries were pooled and sequenced on MiSeq v2 flowcell, with paired-end (PE), 250 bp reads yielding 15.8 M reads (Illumina CSPro lab, Estonian Genome Centre, University of Tartu, Estonia). PE reads were stitched together using MiSeq Reporter software (Illumina, San Diego, USA).

### Raw sequence data processing

Based on our quality assessment, only first set of sequences from the paired group was used in Experiment 1. The raw reads were trimmed from the 3′ end using the program *fastq_quality_trimmer* (parameters: -t 35 -l 200), followed by quality filtering with *fastq_quality_filter* (parameters: -q 30 -p 98) from the FASTX-Toolkit (http://hannonlab.cshl.edu/fastx_toolkit/). PCR primers and sequencing adapters were removed using the program *cutadapt 1.2.1* (parameters: -e 0.2 -n 2 -O 10 -m 100) (https://pypi.python.org/pypi/cutadapt/1.2.1). After trimming, all sequences <100 bp were discarded. In Experiment 2, the first 20 nucleotides from the 5′ end of the stitched raw reads were trimmed using the program *fastx_trimmer* to remove all 8F PCR primer sequences. The reads were converted to their reverse complement sequences and the first 17 nucleotides from the 5′ end were trimmed to remove all 357R primer sequences. Subsequently, the reads were filtered using *fastq_quality_filter* (parameters: -q 30 -p 98), sorted, and written to the file “Pectobacterium“ if they provided a BLASTN hit against the 16S rRNA gene of *P. atrosepticum* SCRI1043 with an identity cutoff of 98% and a match length cutoff of either 100 bp (Experiment 1) or 250 bp (Experiment 2). Chloroplast sequences were removed after comparison with the 16S rRNA gene of *S. tuberosum* isolate DM1-3-516-R44 chloroplast (JF772170) at BLASTN cutoff values of 98% and either 100 bp (Experiment 1) or 250 bp (Experiment 2). All remaining reads with an identity cutoff 98% and match length cutoff of either 100 bp (Experiment 1) or 250 bp (Experiment 2) were classified with RDP *multiclassifier*, an application derived from the RDP Classifier^24^, against the Ribosomal Database Project (RDP) taxonomy training set 9 (trainset9_032012.rdp.fasta) with the default confidence threshold set at 80%. These reads were sorted and written to the file “Other 16S“.

### Sequence processing and assignment of OTUs

All high-quality sequences of the file “Other 16S“ of Experiment 2 were clustered into OTUs and consensus sequences that represented all sequences in that group were derived using AbundantOTU^55^ program (parameters: -abundantonly –d 0.02). Minimum cluster size allowed was five sequences and the lowest intracluster identity allowed was 98%. Single reads that did not belong to any clusters of at least five sequences were clustered again using AbundantOTU with the same parameters as above, however, most of these sequences still did not cluster. All consensus sequences were classified using the RDP classifier^24^ Version 2.8, June 2014. The training set was RDP 16S rRNA training set 9 with a confidence threshold of 80%. In further analysis, a 50% confidence threshold was used to determine the taxonomic position of each read, however, when the confidence of the lowest taxonomic assignment was <50%, the next higher taxon which had at least 50% confidence was used. To avoid chloroplast sequences, all consensus sequences classified as *Streptophyta* were removed from further analysis.

Chimeras were removed using UCHIME^56^ from the mothur^57^ package (chimera.uchime function with parameters: mindiv = 2.0, reference database “rdp gold“ from UCHIME homepage). First, all consensus sequences were analyzed and then all chimeric sequences were removed. All single reads which would not cluster were analyzed and then all chimeras were removed. This resulted in a final set of 294 OTUs and a total of 4,777,229 reads and 3,462 single reads for Experiment 2.

Rarefaction analysis was performed in mothur (rarefaction.single with parameters freq = 10 for samples K1–K6 and freq = 1,000 for other samples).

The final 294 OTUs were identified further by blasting them against a cultivated bacteria dataset using MegaBLAST (parameters: -p 98 -D 3 -v 5 -b 5 -W 52). A top-scoring hit for each consensus with at least 98% of the consensus sequence aligned was chosen as the closest identifiable bacterial strain for each OTU. Because the reads from Experiment 1 are not aligned and correspond to different 100 bp sequences within the V1–V2 hypervariable region, we could not cluster them as in Experiment 2. Instead, we took all 294 OTU consensus sequences from Experiment 2 and then clustered the sequences from Experiment 1 to these using MegaBLAST as above. All reads that had <6 unaligned positions were assigned to the OTU with the highest-scoring match. In total, 539,068 reads were assigned, and of the remaining 180,050 reads, 16,106 were chimeric (using UCHIME as above). The 163,944 unclustered reads were then compared to the cultivated bacteria dataset with MegaBLAST using the above parameters.

### Correlation network analysis

Correlation network analysis was performed to identify the most significant antagonistic and synergistic relationships between bacteria from uninfected potatoes and sets of potatoes infected for a given length of time. We utilized the SparCC method and software developed by Friedman and Alm^58^. To avoid spurious correlations that can arise using data with very small or zero values, we excluded genera that form less than 0.2% of the bacterial population in any given potato or were not detected in more than two potatoes within a given set. After computing the correlations with the main SparCC script (-i 20, -x 10, -t 0.1) we generated bootstrap files using the SparCC script MakeBootstraps (-n 100) and computed correlations for each of these bootstrap replacement files using the main SparCC script with the same parameters. The significance of each correlation was then computed from the bootstrapped results (N = 100) using the SparCC script PseudoPvals. The resulting genera level correlation networks were analyzed using NetworkX (https://networkx.github.io). One network visualization was created for each of the three sets of potatoes from Experiment 2 (uninfected, two days post-infection, and eight days post-infection) using matplotlib (http://matplotlib.org) and combined to create Fig. 7.

## Additional Information

**How to cite this article**: Kõiv, V. *et al.* Microbial population dynamics in response to *Pectobacterium atrosepticum* infection in potato tubers. *Sci. Rep.* **5**, 11606; doi: 10.1038/srep11606 (2015).

## Supplementary Material

Supplementary Information
