# Supplementary material for: Microbial population dynamics in response to Pectobacterium atrosepticum infection in potato tubers
Source: Sci Rep. 2015 Jun 29;5:11606. doi: 10.1038/srep11606 (PMC4484245; doi:10.1038/srep11606)
Supplement: Supplementary Information [file srep11606-s1.pdf]

# Microbial population dynamics in response to *Pectobacterium atrosepticum* infection in potato tubers

**Viia Kõiv*[1], Märt Roosaare[1], Eve Vedler[1], Paula Ann Kivistik[2], Kristel Toppi[1], David W. Schryer[3], Maido Remm[1], Tanel Tenson[3], Andres Mäe[1]**

*Institute of Molecular and Cell Biology, University of Tartu, 23 Riia Street, Tartu 51010, Estonia[1]*

*Estonian Genome Center, University of Tartu, Riia 23 B, 51010 Tartu, Estonia[2]*

*Institute of Technology, University of Tartu, 1 Nooruse Street, Tartu 50411, Estonia[3]*

E-mail: viia.koiv@ut.ee

# Supplemental Materials:

**Supplemental info.** Technical aspects of potato var. “Ants” cultivation.

**Supplemental Data.** OUTs generated based on sequencing reads of Experiment 2.

**Supplemental Figure S1.** Heatmaps of Experiment 1.

**Supplemental Figure S2.** Phylogenetic tree of cultivable bacteria based on the 16S rRNA gene sequence comparison.

**Potato cultivar „Ants“ 2012:**

Cultivation area 0.5 ha. Planted Basic seeds 2000kg/ha.

Mineral soil, loam

Preceding crops: 2009 summer wheat, 2010 rape, 2011 summer wheat.

Seed dressing: Monceren G, 0.6 l/ha.

Planting time: 17.05. 2012, harvesting 06.09.2012, crop yield 26.0 t/ha.

Fertilizer: Cropcare 8-11-23 800kg/ha 10.05.2012

Plant protection:

1. 21.06. 2012 Titus 30 g/ha+Mistral 300 g/ha+Actara 80 g/ha
2. 09.07.2012 Dithane 2 kg/ha+ Decis 0.15 l/ha+Kemiwett 0.15 l/ha
3. 17.07.2012 Leimay 0.5 l/ha+Actara 60 g/ha+Kemiwett 0.15 l/ha
4. 24.07. 2012 Infinito 1.6 l/ha
5. 11.08.2012 Ranman 0.2 l/ha+Ranman Aktivaator 0.15 l/ha+Decis 0.15 l/ha+Kemiwett 0.15 l/ha
6. 13.08.2012 Sunoco E11 oil 4,
7. 17.08.2012 Reglone Super 2.0 l/ha

**Potato cultivar „Ants“ 2013:**

Cultivation area 0.5 ha. Planted Basic seeds 5000 kg/ha

Mineral soil, loam

Preceding crops: 2010 barley, 2011 barley, 2012 rape.

Seeds not dressed.

Planting time: 27.05. 2012, harvesting 05.09.2012, crop yield 29.0 t/ha.

Fertilizer: Cropcare 11-11-21 800kg/ha 09.05.2012

Plant protection:

1. 19.06. 2013 Titus 30 g/ha+Mistral 300 g/ha+ Decis 0.15 l/ha
2. 01.07.2013 Dithane 2 kg/ha+ Fastac 0.15 l/ha+Titus 30 g/ha
3. 09.07.2013 Infinito 1.6 l/ha+Decis Mega 0.15 l/ha
4. 10.07.2013 Sunoco E11/3
5. 18.07.2013 Dithane 2.0 kg/ha+ Fastac 0.15 l/ha
6. 24.07.2013 Sunoco E11/3 4.0 l/ha
7. 29.07.2013 Orwego 0.8 l/ha + Kemiwett 0,075 l/ha
8. 06.08.2013 Orwego 0.8 l/ha+ Kemiwett 0,075 l/ha
9. 13.08. 2013 Reglone Super 2.5 l/ha+ Kemiwett 0,075 l

**Supplemental info. Technical aspects of potato var. “Ants” cultivation.**

A

| OTU | K1 | K2 | K3 | K4 | K5 | K6 | 2d2 | 2d6 | 2d7 | 2d10 | 2d11 | 2d12 | 2d13 | 8d1 | 8d3 | 8d8 | 8d11 | 8d12 | 8d13 |
|---|---|---|---|---|---|---|---|---|---|---|---|---|---|---|---|---|---|---|---|
| Bosea | 1 | 0 | 1 | 0 | 0 | 0 | 0 | 0 | 0 | 1 | 0 | 1 | 0 | 14 | 4 | 1 | 217 | 19 | 13 |
| Consensus156 | 1 | 0 | 1 | 0 | 0 | 0 | 0 | 0 | 0 | 1 | 0 | 1 | 0 | 14 | 4 | 1 | 217 | 19 | 13 |
| Planococcaceae | 0 | 0 | 0 | 0 | 0 | 0 | 0 | 0 | 0 | 0 | 0 | 0 | 0 | 0 | 0 | 0 | 0 | 0 | 7 |
| Consensus437 | 0 | 0 | 0 | 0 | 0 | 0 | 0 | 0 | 0 | 0 | 0 | 0 | 0 | 0 | 0 | 0 | 0 | 0 | 7 |
| Methylobacillus | 0 | 0 | 0 | 0 | 0 | 0 | 0 | 0 | 0 | 0 | 0 | 0 | 0 | 29 | 1 | 0 | 0 | 77 | 0 |
| Consensus242 | 0 | 0 | 0 | 0 | 0 | 0 | 0 | 0 | 0 | 0 | 0 | 0 | 0 | 29 | 1 | 0 | 0 | 77 | 0 |
| Pantoea | 0 | 0 | 0 | 0 | 0 | 19 | 134 | 438 | 1770 | 246 | 38833 | 768 | 2 | 0 | 43 | 44 | 85 | 16 | 29 |
| Consensus273 | 0 | 0 | 0 | 0 | 0 | 0 | 1 | 0 | 7 | 0 | 74 | 4 | 0 | 0 | 1 | 0 | 0 | 0 | 0 |
| Consensus194 | 0 | 0 | 0 | 0 | 0 | 0 | 0 | 5 | 40 | 0 | 133 | 5 | 0 | 0 | 0 | 0 | 0 | 0 | 0 |
| Consensus40 | 0 | 0 | 0 | 0 | 0 | 2 | 18 | 52 | 165 | 18 | 5043 | 64 | 0 | 0 | 8 | 4 | 12 | 3 | 3 |
| Consensus15 | 0 | 0 | 0 | 0 | 0 | 17 | 115 | 381 | 1558 | 228 | 33583 | 695 | 2 | 0 | 34 | 40 | 73 | 13 | 26 |
| Bacillus | 0 | 0 | 0 | 0 | 0 | 0 | 4044 | 439 | 7976 | 4813 | 305 | 186 | 168 | 63 | 28 | 94 | 33 | 140 | 29 |
| Consensus436 | 0 | 0 | 0 | 0 | 0 | 0 | 0 | 0 | 7 | 0 | 0 | 0 | 0 | 0 | 0 | 0 | 0 | 0 | 0 |
| Consensus407 | 0 | 0 | 0 | 0 | 0 | 0 | 1 | 0 | 8 | 10 | 0 | 1 | 0 | 0 | 0 | 0 | 0 | 0 | 0 |
| Consensus405 | 0 | 0 | 0 | 0 | 0 | 0 | 0 | 0 | 20 | 0 | 0 | 1 | 0 | 0 | 0 | 0 | 0 | 0 | 0 |
| Consensus151 | 0 | 0 | 0 | 0 | 0 | 0 | 0 | 4 | 0 | 0 | 0 | 1 | 0 | 58 | 22 | 88 | 0 | 140 | 0 |
| Consensus105 | 0 | 0 | 0 | 0 | 0 | 0 | 206 | 159 | 95 | 189 | 23 | 1 | 22 | 5 | 0 | 0 | 6 | 0 | 0 |
| Consensus45 | 0 | 0 | 0 | 0 | 0 | 0 | 3 | 4 | 3450 | 5 | 173 | 52 | 77 | 0 | 3 | 0 | 11 | 0 | 29 |
| Consensus26 | 0 | 0 | 0 | 0 | 0 | 0 | 3834 | 272 | 4396 | 4609 | 109 | 130 | 69 | 0 | 3 | 6 | 16 | 0 | 0 |
| Pectobacterium | 9 | 2 | 8 | 2 | 2 | 3 | 922 | 2525 | 3701 | 750 | 1143 | 1269 | 181 | 2967 | 1704 | 2174 | 10137 | 5432 | 10108 |
| Consensus370 | 0 | 0 | 0 | 0 | 0 | 0 | 0 | 0 | 0 | 0 | 0 | 0 | 0 | 0 | 1 | 1 | 0 | 0 | 30 |
| Consensus353 | 0 | 0 | 0 | 0 | 0 | 0 | 0 | 14 | 8 | 0 | 1 | 5 | 0 | 0 | 2 | 2 | 0 | 7 | 0 |
| Consensus348 | 0 | 0 | 0 | 0 | 0 | 0 | 0 | 0 | 1 | 0 | 2 | 2 | 0 | 17 | 7 | 3 | 2 | 6 | 0 |
| Consensus325 | 0 | 0 | 0 | 0 | 0 | 0 | 3 | 11 | 3 | 4 | 0 | 16 | 0 | 0 | 0 | 0 | 2 | 10 | 1 |
| Consensus321 | 0 | 0 | 0 | 0 | 0 | 0 | 0 | 4 | 19 | 2 | 2 | 0 | 0 | 0 | 1 | 2 | 0 | 21 | 0 |
| Consensus312 | 0 | 0 | 0 | 0 | 0 | 0 | 4 | 1 | 1 | 3 | 0 | 1 | 0 | 2 | 9 | 14 | 14 | 8 | 0 |
| Consensus293 | 0 | 0 | 0 | 0 | 0 | 0 | 2 | 22 | 10 | 0 | 0 | 6 | 0 | 5 | 4 | 12 | 0 | 7 | 0 |
| Consensus211 | 0 | 0 | 0 | 0 | 0 | 0 | 18 | 28 | 17 | 15 | 16 | 12 | 0 | 10 | 2 | 17 | 9 | 12 | 1 |
| Consensus46 | 7 | 2 | 5 | 1 | 0 | 1 | 537 | 227 | 139 | 383 | 399 | 134 | 26 | 332 | 343 | 194 | 562 | 364 | 117 |
| Consensus29 | 2 | 0 | 0 | 0 | 2 | 2 | 261 | 2176 | 3499 | 206 | 676 | 1063 | 38 | 244 | 160 | 571 | 121 | 2291 | 17 |
| Consensus17 | 0 | 0 | 3 | 1 | 0 | 0 | 97 | 42 | 4 | 137 | 47 | 30 | 117 | 2357 | 1175 | 1358 | 9427 | 2706 | 9942 |
| Agromyces | 0 | 0 | 0 | 0 | 0 | 0 | 0 | 0 | 0 | 0 | 0 | 1 | 0 | 43 | 1 | 0 | 17 | 47 | 107 |
| Consensus177 | 0 | 0 | 0 | 0 | 0 | 0 | 0 | 0 | 0 | 0 | 0 | 1 | 0 | 43 | 1 | 0 | 17 | 47 | 107 |
| Massilia | 0 | 0 | 0 | 0 | 0 | 0 | 6 | 628 | 6 | 1 | 44 | 117 | 0 | 44 | 16 | 1 | 33 | 1 | 4 |
| Consensus426 | 0 | 0 | 0 | 0 | 0 | 0 | 0 | 8 | 0 | 0 | 1 | 0 | 0 | 1 | 0 | 0 | 0 | 0 | 0 |
| Consensus382 | 0 | 0 | 0 | 0 | 0 | 0 | 1 | 2 | 4 | 1 | 0 | 16 | 0 | 5 | 0 | 0 | 0 | 0 | 0 |
| Consensus91 | 0 | 0 | 0 | 0 | 0 | 0 | 5 | 618 | 2 | 0 | 43 | 101 | 0 | 38 | 16 | 1 | 33 | 1 | 4 |
| Domibacillus | 0 | 0 | 0 | 0 | 0 | 0 | 0 | 14 | 0 | 0 | 0 | 0 | 0 | 0 | 0 | 0 | 0 | 0 | 0 |
| Consensus419 | 0 | 0 | 0 | 0 | 0 | 0 | 0 | 14 | 0 | 0 | 0 | 0 | 0 | 0 | 0 | 0 | 0 | 0 | 0 |
| Dyadobacter | 0 | 0 | 0 | 0 | 0 | 0 | 2 | 0 | 0 | 1 | 0 | 0 | 0 | 25 | 0 | 3 | 48 | 1 | 0 |
| Consensus371 | 0 | 0 | 0 | 0 | 0 | 0 | 2 | 0 | 0 | 1 | 0 | 0 | 0 | 21 | 0 | 3 | 4 | 1 | 0 |
| Consensus327 | 0 | 0 | 0 | 0 | 0 | 0 | 0 | 0 | 0 | 0 | 0 | 0 | 0 | 4 | 0 | 0 | 44 | 0 | 0 |
| Devosia | 0 | 1 | 5 | 0 | 0 | 8 | 1 | 1 | 1 | 0 | 0 | 1 | 0 | 190 | 300 | 8 | 263 | 125 | 45 |
| Consensus174 | 0 | 1 | 2 | 0 | 0 | 7 | 1 | 1 | 1 | 0 | 0 | 1 | 0 | 44 | 59 | 7 | 66 | 24 | 7 |
| Consensus102 | 0 | 0 | 3 | 0 | 0 | 1 | 0 | 0 | 0 | 0 | 0 | 0 | 0 | 146 | 241 | 1 | 197 | 101 | 38 |
| Enterobacter | 70 | 7 | 39 | 20 | 0 | 31 | 1E+05 | 44712 | 15594 | 1E+05 | 24556 | 34240 | 36124 | 2E+05 | 36066 | 23571 | 2E+05 | 32517 | 3E+05 |
| Consensus221 | 0 | 0 | 0 | 0 | 0 | 0 | 1 | 22 | 0 | 0 | 1 | 0 | 0 | 7 | 11 | 20 | 14 | 14 | 41 |
| Consensus2 | 70 | 7 | 39 | 20 | 0 | 31 | 1E+05 | 44690 | 15594 | 1E+05 | 24555 | 34240 | 36124 | 2E+05 | 36055 | 23551 | 2E+05 | 32503 | 3E+05 |
| Stenotrophomonas | 6 | 0 | 1 | 0 | 0 | 53 | 13184 | 19429 | 10356 | 17044 | 2762 | 15735 | 2256 | 46991 | 12470 | 65563 | 67384 | 16689 | 6761 |
| Consensus417 | 0 | 0 | 0 | 0 | 0 | 0 | 2 | 1 | 1 | 0 | 0 | 4 | 0 | 0 | 5 | 1 | 1 | 0 | 0 |
| Consensus384 | 0 | 0 | 0 | 0 | 0 | 0 | 5 | 3 | 5 | 0 | 0 | 5 | 0 | 5 | 3 | 1 | 1 | 0 | 0 |
| Consensus343 | 0 | 0 | 0 | 0 | 0 | 0 | 0 | 0 | 0 | 0 | 0 | 0 | 0 | 8 | 0 | 0 | 13 | 20 | 0 |
| Consensus341 | 0 | 0 | 0 | 0 | 0 | 0 | 7 | 2 | 2 | 6 | 0 | 2 | 0 | 3 | 0 | 6 | 0 | 14 | 0 |
| Consensus334 | 0 | 0 | 0 | 0 | 0 | 1 | 8 | 0 | 0 | 1 | 0 | 1 | 0 | 0 | 10 | 4 | 2 | 17 | 0 |
| Consensus317 | 0 | 0 | 0 | 0 | 0 | 0 | 1 | 0 | 0 | 0 | 0 | 1 | 0 | 7 | 7 | 11 | 17 | 7 | 2 |
| Consensus304 | 0 | 0 | 0 | 0 | 0 | 0 | 1 | 2 | 17 | 5 | 0 | 0 | 0 | 1 | 2 | 16 | 1 | 13 | 4 |
| Consensus268 | 0 | 0 | 0 | 0 | 0 | 0 | 0 | 22 | 26 | 1 | 0 | 8 | 1 | 1 | 2 | 22 | 0 | 7 | 0 |
| Consensus219 | 0 | 0 | 0 | 0 | 0 | 2 | 13 | 2 | 0 | 16 | 1 | 11 | 0 | 23 | 9 | 30 | 25 | 2 | 3 |
| Consensus216 | 0 | 0 | 0 | 0 | 0 | 0 | 0 | 0 | 0 | 0 | 0 | 2 | 0 | 18 | 0 | 0 | 86 | 35 | 1 |
| Consensus148 | 0 | 0 | 0 | 0 | 0 | 0 | 0 | 0 | 0 | 0 | 0 | 1 | 0 | 18 | 0 | 0 | 44 | 0 | 273 |
| Consensus116 | 0 | 0 | 0 | 0 | 0 | 0 | 15 | 110 | 170 | 10 | 6 | 45 | 0 | 34 | 19 | 147 | 0 | 94 | 0 |
| Consensus85 | 0 | 0 | 0 | 0 | 0 | 0 | 44 | 68 | 455 | 54 | 1 | 1 | 3 | 6 | 12 | 72 | 91 | 261 | 29 |
| Consensus66 | 0 | 0 | 1 | 0 | 0 | 3 | 85 | 164 | 3 | 106 | 4 | 100 | 9 | 22 | 544 | 85 | 93 | 626 | 19 |
| Consensus59 | 0 | 0 | 0 | 0 | 0 | 0 | 125 | 334 | 258 | 119 | 44 | 336 | 2 | 324 | 142 | 221 | 404 | 83 | 25 |
| Consensus13 | 0 | 0 | 0 | 0 | 0 | 0 | 0 | 0 | 0 | 1 | 2 | 184 | 0 | 3414 | 19 | 26 | 23607 | 7883 | 4205 |
| Consensus6 | 6 | 0 | 0 | 0 | 0 | 47 | 12878 | 18721 | 9419 | 16725 | 2704 | 15034 | 2241 | 43107 | 11696 | 64921 | 42999 | 7627 | 2200 |
| Pelosinus | 0 | 0 | 0 | 0 | 0 | 0 | 0 | 0 | 0 | 0 | 0 | 0 | 0 | 3 | 2 | 3 | 0 | 138 | 11 |
| Consensus210 | 0 | 0 | 0 | 0 | 0 | 0 | 0 | 0 | 0 | 0 | 0 | 0 | 0 | 3 | 2 | 3 | 0 | 138 | 11 |
| Duganella | 0 | 0 | 0 | 0 | 0 | 0 | 4 | 13 | 0 | 1 | 0 | 22 | 3 | 2 | 0 | 0 | 6 | 0 | 0 |
| Consensus322 | 0 | 0 | 0 | 0 | 0 | 0 | 4 | 13 | 0 | 1 | 0 | 22 | 3 | 2 | 0 | 0 | 6 | 0 | 0 |

| | | | | | | | | | | | | | | | | | | | |
|---|---|---|---|---|---|---|---|---|---|---|---|---|---|---|---|---|---|---|---|
| Achromobacter | 4 | 3 | 14 | 4 | 4 | 7 | 437 | 990 | 1383 | 423 | 472 | 633 | 21 | 17182 | 5350 | 3330 | 12041 | 11348 | 2496 |
| Consensus10 | 4 | 3 | 14 | 4 | 4 | 7 | 437 | 990 | 1383 | 423 | 472 | 633 | 21 | 17182 | 5350 | 3330 | 12041 | 11348 | 2496 |
| Zymophilus | 0 | 0 | 0 | 0 | 0 | 0 | 0 | 0 | 0 | 0 | 0 | 0 | 0 | 0 | 18 | 0 | 0 | 0 | 0 |
| Consensus410 | 0 | 0 | 0 | 0 | 0 | 0 | 0 | 0 | 0 | 0 | 0 | 0 | 0 | 0 | 18 | 0 | 0 | 0 | 0 |
| Comamonas | 16 | 0 | 6 | 0 | 0 | 1 | 5890 | 883 | 1 | 6029 | 779 | 809 | 537 | 22470 | 22882 | 2823 | 75072 | 1E+05 | 99595 |
| Consensus400 | 0 | 0 | 0 | 0 | 0 | 0 | 0 | 0 | 0 | 0 | 0 | 0 | 0 | 0 | 7 | 0 | 9 | 1 | 5 |
| Consensus380 | 0 | 0 | 0 | 0 | 0 | 0 | 8 | 0 | 0 | 7 | 0 | 0 | 0 | 8 | 1 | 0 | 4 | 1 | 0 |
| Consensus254 | 0 | 0 | 0 | 0 | 0 | 0 | 0 | 0 | 0 | 0 | 0 | 0 | 0 | 2 | 21 | 0 | 18 | 34 | 23 |
| Consensus237 | 0 | 0 | 0 | 0 | 0 | 0 | 7 | 0 | 0 | 6 | 0 | 0 | 0 | 30 | 11 | 0 | 23 | 22 | 12 |
| Consensus185 | 0 | 0 | 0 | 0 | 0 | 0 | 0 | 0 | 0 | 0 | 0 | 0 | 0 | 0 | 0 | 17 | 180 | 0 | 0 |
| Consensus23 | 10 | 0 | 0 | 0 | 0 | 0 | 0 | 0 | 0 | 0 | 1 | 0 | 1 | 661 | 14682 | 203 | 35 | 580 | 0 |
| Consensus8 | 2 | 0 | 2 | 0 | 0 | 0 | 5863 | 20 | 0 | 5982 | 637 | 441 | 140 | 18147 | 2841 | 841 | 31752 | 2424 | 8251 |
| Consensus5 | 4 | 0 | 4 | 0 | 0 | 1 | 12 | 863 | 1 | 34 | 141 | 368 | 396 | 3622 | 5319 | 1762 | 43051 | 1E+05 | 91304 |
| Shinella | 0 | 0 | 0 | 0 | 0 | 1 | 1 | 3 | 5 | 0 | 0 | 5 | 0 | 4 | 0 | 0 | 3 | 4 | 0 |
| Consensus391 | 0 | 0 | 0 | 0 | 0 | 1 | 1 | 3 | 5 | 0 | 0 | 5 | 0 | 4 | 0 | 0 | 3 | 4 | 0 |
| Lysinibacillus | 0 | 0 | 0 | 0 | 0 | 0 | 0 | 0 | 0 | 4 | 19 | 3 | 0 | 0 | 0 | 4 | 369 | 14444 | 5400 |
| Consensus432 | 0 | 0 | 0 | 0 | 0 | 0 | 0 | 0 | 0 | 0 | 0 | 1 | 0 | 0 | 0 | 0 | 0 | 7 | 0 |
| Consensus379 | 0 | 0 | 0 | 0 | 0 | 0 | 0 | 0 | 0 | 0 | 0 | 0 | 0 | 0 | 0 | 0 | 0 | 29 | 0 |
| Consensus345 | 0 | 0 | 0 | 0 | 0 | 0 | 0 | 0 | 0 | 0 | 0 | 0 | 0 | 0 | 0 | 0 | 0 | 0 | 41 |
| Consensus284 | 0 | 0 | 0 | 0 | 0 | 0 | 0 | 0 | 0 | 0 | 0 | 0 | 0 | 0 | 0 | 0 | 0 | 80 | 0 |
| Consensus154 | 0 | 0 | 0 | 0 | 0 | 0 | 0 | 0 | 0 | 0 | 0 | 0 | 0 | 0 | 0 | 0 | 0 | 0 | 284 |
| Consensus144 | 0 | 0 | 0 | 0 | 0 | 0 | 0 | 0 | 0 | 0 | 0 | 0 | 0 | 0 | 0 | 0 | 368 | 0 | 0 |
| Consensus42 | 0 | 0 | 0 | 0 | 0 | 0 | 0 | 0 | 0 | 2 | 0 | 2 | 0 | 0 | 0 | 0 | 1 | 0 | 5075 |
| Consensus25 | 0 | 0 | 0 | 0 | 0 | 0 | 0 | 0 | 0 | 2 | 19 | 0 | 0 | 0 | 0 | 4 | 0 | 14328 | 0 |
| Paracoccus | 0 | 0 | 0 | 0 | 0 | 0 | 0 | 12 | 0 | 0 | 0 | 0 | 0 | 35 | 0 | 0 | 75 | 0 | 1 |
| Consensus413 | 0 | 0 | 0 | 0 | 0 | 0 | 0 | 12 | 0 | 0 | 0 | 0 | 0 | 0 | 0 | 0 | 4 | 0 | 0 |
| Consensus241 | 0 | 0 | 0 | 0 | 0 | 0 | 0 | 0 | 0 | 0 | 0 | 0 | 0 | 35 | 0 | 0 | 71 | 0 | 1 |
| Enterobacteriaceae | 0 | 0 | 0 | 0 | 0 | 0 | 838 | 452 | 199 | 595 | 96 | 268 | 34 | 2874 | 431 | 749 | 2296 | 697 | 1124 |
| Consensus435 | 0 | 0 | 0 | 0 | 0 | 0 | 0 | 0 | 2 | 0 | 0 | 0 | 0 | 0 | 0 | 2 | 3 | 1 | 0 |
| Consensus378 | 0 | 0 | 0 | 0 | 0 | 0 | 1 | 6 | 1 | 0 | 0 | 0 | 0 | 4 | 3 | 4 | 0 | 10 | 0 |
| Consensus326 | 0 | 0 | 0 | 0 | 0 | 0 | 4 | 0 | 0 | 4 | 0 | 1 | 0 | 16 | 1 | 11 | 5 | 3 | 3 |
| Consensus289 | 0 | 0 | 0 | 0 | 0 | 0 | 1 | 27 | 2 | 0 | 16 | 6 | 0 | 3 | 5 | 4 | 2 | 8 | 1 |
| Consensus196 | 0 | 0 | 0 | 0 | 0 | 0 | 24 | 22 | 26 | 5 | 5 | 13 | 2 | 12 | 13 | 8 | 0 | 43 | 5 |
| Consensus175 | 0 | 0 | 0 | 0 | 0 | 0 | 14 | 5 | 0 | 6 | 2 | 3 | 0 | 96 | 14 | 22 | 37 | 10 | 10 |
| Consensus126 | 0 | 0 | 0 | 0 | 0 | 0 | 31 | 31 | 24 | 18 | 6 | 30 | 0 | 92 | 17 | 137 | 87 | 31 | 0 |
| Consensus54 | 0 | 0 | 0 | 0 | 0 | 0 | 763 | 340 | 142 | 561 | 48 | 212 | 28 | 316 | 98 | 92 | 365 | 28 | 19 |
| Consensus37 | 0 | 0 | 0 | 0 | 0 | 0 | 0 | 21 | 2 | 1 | 19 | 3 | 4 | 2335 | 280 | 469 | 1797 | 563 | 1086 |
| Ensifer | 0 | 1 | 0 | 2 | 0 | 0 | 1 | 14 | 0 | 0 | 0 | 3 | 0 | 6 | 1 | 0 | 8 | 8 | 9 |
| Consensus319 | 0 | 1 | 0 | 2 | 0 | 0 | 1 | 14 | 0 | 0 | 0 | 3 | 0 | 6 | 1 | 0 | 8 | 8 | 9 |
| Buttiauxella | 0 | 0 | 0 | 0 | 0 | 0 | 14 | 16 | 1 | 10 | 1 | 1 | 0 | 50 | 12 | 1 | 18 | 1 | 3 |
| Consensus223 | 0 | 0 | 0 | 0 | 0 | 0 | 14 | 16 | 1 | 10 | 1 | 1 | 0 | 50 | 12 | 1 | 18 | 1 | 3 |
| Rhodococcus | 0 | 0 | 0 | 0 | 0 | 0 | 0 | 0 | 0 | 0 | 0 | 0 | 0 | 0 | 0 | 0 | 5 | 87 | 0 |
| Consensus259 | 0 | 0 | 0 | 0 | 0 | 0 | 0 | 0 | 0 | 0 | 0 | 0 | 0 | 0 | 0 | 0 | 5 | 87 | 0 |
| Rhizobium | 0 | 2 | 26 | 0 | 0 | 122 | 555 | 2001 | 1610 | 627 | 247 | 531 | 223 | 8195 | 3611 | 1774 | 4469 | 3338 | 2360 |
| Consensus333 | 0 | 0 | 0 | 0 | 0 | 0 | 1 | 3 | 0 | 1 | 2 | 0 | 0 | 0 | 0 | 0 | 30 | 7 | 0 |
| Consensus309 | 0 | 0 | 0 | 0 | 0 | 0 | 0 | 2 | 4 | 0 | 0 | 2 | 0 | 14 | 29 | 0 | 1 | 4 | 2 |
| Consensus142 | 0 | 0 | 0 | 0 | 0 | 0 | 24 | 12 | 75 | 56 | 2 | 9 | 1 | 77 | 9 | 5 | 87 | 16 | 3 |
| Consensus140 | 0 | 0 | 0 | 0 | 0 | 0 | 2 | 19 | 16 | 0 | 2 | 16 | 0 | 29 | 226 | 5 | 16 | 49 | 15 |
| Consensus112 | 0 | 0 | 0 | 0 | 0 | 0 | 1 | 8 | 0 | 1 | 0 | 1 | 0 | 5 | 1 | 380 | 129 | 155 | 11 |
| Consensus104 | 0 | 0 | 0 | 0 | 0 | 0 | 15 | 118 | 125 | 14 | 17 | 85 | 22 | 24 | 44 | 31 | 197 | 13 | 11 |
| Consensus38 | 0 | 1 | 14 | 0 | 0 | 121 | 147 | 428 | 846 | 122 | 58 | 104 | 14 | 1177 | 802 | 363 | 823 | 466 | 501 |
| Consensus21 | 0 | 1 | 12 | 0 | 0 | 1 | 365 | 1411 | 544 | 433 | 166 | 314 | 186 | 6869 | 2500 | 990 | 3186 | 2628 | 1817 |
| Propionibacterium | 21 | 12 | 17 | 56 | 33 | 37 | 9 | 21 | 61 | 18 | 17 | 27 | 3 | 25 | 8 | 4 | 4 | 12 | 15 |
| Consensus138 | 21 | 12 | 17 | 56 | 33 | 37 | 9 | 21 | 61 | 18 | 17 | 27 | 3 | 25 | 8 | 4 | 4 | 12 | 15 |
| Clostridium XlVa | 0 | 0 | 1 | 0 | 0 | 0 | 0 | 2 | 0 | 1 | 0 | 0 | 0 | 247 | 8167 | 2726 | 11410 | 28 | 36665 |
| Consensus402 | 0 | 0 | 0 | 0 | 0 | 0 | 0 | 0 | 0 | 0 | 0 | 0 | 0 | 1 | 0 | 0 | 0 | 0 | 21 |
| Consensus52 | 0 | 0 | 0 | 0 | 0 | 0 | 0 | 0 | 0 | 0 | 0 | 0 | 0 | 235 | 1 | 2596 | 275 | 26 | 166 |
| Consensus11 | 0 | 0 | 1 | 0 | 0 | 0 | 0 | 2 | 0 | 1 | 0 | 0 | 0 | 11 | 8166 | 130 | 11135 | 2 | 36478 |
| Variovorax | 1 | 2 | 14 | 4 | 0 | 40 | 14 | 30 | 3 | 11 | 1 | 6 | 0 | 454 | 22 | 3 | 443 | 35 | 1 |
| Consensus86 | 1 | 2 | 14 | 4 | 0 | 40 | 14 | 30 | 3 | 11 | 1 | 6 | 0 | 454 | 22 | 3 | 443 | 35 | 1 |
| Phyllobacterium | 0 | 0 | 17 | 0 | 0 | 0 | 3 | 5 | 4 | 0 | 0 | 1 | 0 | 79 | 25 | 0 | 11 | 2 | 0 |
| Consensus213 | 0 | 0 | 17 | 0 | 0 | 0 | 3 | 5 | 4 | 0 | 0 | 1 | 0 | 79 | 25 | 0 | 11 | 2 | 0 |
| Sphingobium | 0 | 0 | 9 | 0 | 0 | 6 | 3 | 24 | 15 | 5 | 3 | 26 | 0 | 35 | 0 | 0 | 78 | 0 | 0 |
| Consensus279 | 0 | 0 | 9 | 0 | 0 | 6 | 1 | 17 | 0 | 0 | 2 | 2 | 0 | 12 | 0 | 0 | 35 | 0 | 0 |
| Consensus226 | 0 | 0 | 0 | 0 | 0 | 0 | 2 | 7 | 15 | 5 | 1 | 24 | 0 | 23 | 0 | 0 | 43 | 0 | 0 |
| Clostridium sensu stricto | 0 | 0 | 0 | 0 | 0 | 0 | 0 | 0 | 0 | 17 | 47 | 0 | 1 | 11 | 0 | 313 | 1 | 0 | 0 |
| Consensus425 | 0 | 0 | 0 | 0 | 0 | 0 | 0 | 0 | 0 | 0 | 0 | 0 | 0 | 6 | 0 | 3 | 1 | 0 | 0 |
| Consensus197 | 0 | 0 | 0 | 0 | 0 | 0 | 0 | 0 | 0 | 0 | 0 | 0 | 0 | 5 | 0 | 172 | 0 | 0 | 0 |
| Consensus181 | 0 | 0 | 0 | 0 | 0 | 0 | 0 | 0 | 0 | 17 | 47 | 0 | 1 | 0 | 0 | 138 | 0 | 0 | 0 |
| Microbacterium | 0 | 0 | 1 | 0 | 0 | 5 | 6 | 30 | 2 | 4 | 3 | 30 | 2 | 128 | 47 | 5 | 256 | 110 | 28 |
| Consensus433 | 0 | 0 | 0 | 0 | 0 | 0 | 0 | 3 | 0 | 0 | 0 | 0 | 1 | 4 | 0 | 0 | 0 | 0 | 0 |
| Consensus418 | 0 | 0 | 0 | 0 | 0 | 1 | 0 | 0 | 0 | 0 | 1 | 0 | 0 | 0 | 0 | 0 | 3 | 10 | 0 |
| Consensus403 | 0 | 0 | 1 | 0 | 0 | 0 | 0 | 0 | 0 | 0 | 0 | 0 | 0 | 3 | 0 | 0 | 17 | 0 | 0 |
| Consensus374 | 0 | 0 | 0 | 0 | 0 | 0 | 0 | 6 | 0 | 0 | 2 | 1 | 0 | 5 | 1 | 0 | 14 | 2 | 0 |

| | | | | | | | | | | | | | | | | | | | |
|---|---|---|---|---|---|---|---|---|---|---|---|---|---|---|---|---|---|---|---|
| Consensus245 | 0 | 0 | 0 | 0 | 0 | 0 | 0 | 0 | 0 | 2 | 0 | 2 | 0 | 7 | 1 | 0 | 55 | 33 | 4 |
| Consensus232 | 0 | 0 | 0 | 0 | 0 | 0 | 0 | 0 | 0 | 0 | 0 | 1 | 0 | 9 | 2 | 0 | 87 | 11 | 3 |
| Consensus195 | 0 | 0 | 0 | 0 | 0 | 1 | 5 | 5 | 0 | 2 | 0 | 21 | 0 | 16 | 8 | 0 | 67 | 37 | 19 |
| Consensus193 | 0 | 0 | 0 | 0 | 0 | 3 | 1 | 16 | 2 | 0 | 0 | 5 | 1 | 84 | 35 | 5 | 13 | 17 | 2 |
| Delftia | 22 | 1 | 4 | 11 | 0 | 1 | 149 | 5880 | 51 | 144 | 204 | 1007 | 169 | 28948 | 25584 | 2E+05 | 97954 | 78201 | 17899 |
| Consensus415 | 0 | 0 | 0 | 0 | 0 | 0 | 0 | 0 | 0 | 0 | 0 | 0 | 0 | 3 | 6 | 0 | 5 | 1 | 1 |
| Consensus390 | 0 | 0 | 0 | 0 | 0 | 0 | 0 | 0 | 0 | 0 | 0 | 0 | 0 | 4 | 2 | 2 | 15 | 1 | 2 |
| Consensus362 | 0 | 0 | 0 | 0 | 0 | 0 | 0 | 1 | 0 | 0 | 0 | 0 | 0 | 1 | 19 | 5 | 7 | 1 | 0 |
| Consensus3 | 22 | 1 | 4 | 11 | 0 | 1 | 149 | 5879 | 51 | 144 | 204 | 1007 | 169 | 28940 | 25557 | 2E+05 | 97927 | 78198 | 17896 |
| Pseudorhodoferax | 0 | 0 | 0 | 0 | 0 | 0 | 0 | 12 | 1 | 4 | 0 | 4 | 0 | 24 | 3 | 0 | 379 | 11 | 8 |
| Consensus134 | 0 | 0 | 0 | 0 | 0 | 0 | 0 | 12 | 1 | 4 | 0 | 4 | 0 | 24 | 3 | 0 | 379 | 11 | 8 |
| Herminiimonas | 0 | 0 | 18 | 0 | 0 | 3 | 0 | 3 | 0 | 5 | 0 | 1 | 0 | 826 | 40 | 67 | 463 | 410 | 80 |
| Consensus421 | 0 | 0 | 2 | 0 | 0 | 0 | 0 | 0 | 0 | 0 | 0 | 0 | 0 | 2 | 1 | 0 | 2 | 6 | 1 |
| Consensus65 | 0 | 0 | 16 | 0 | 0 | 3 | 0 | 3 | 0 | 5 | 0 | 1 | 0 | 824 | 39 | 67 | 461 | 404 | 79 |
| Yersinia | 0 | 0 | 0 | 0 | 0 | 0 | 1919 | 6728 | 8421 | 3339 | 7 | 1251 | 1711 | 320 | 1 | 282 | 6051 | 3495 | 2857 |
| Consensus16 | 0 | 0 | 0 | 0 | 0 | 0 | 1919 | 6728 | 8421 | 3339 | 7 | 1251 | 1711 | 320 | 1 | 282 | 6051 | 3495 | 2857 |
| Serratia | 1 | 15 | 6 | 6 | 4 | 5 | 23801 | 4567 | 13261 | 24849 | 5307 | 1077 | 3014 | 4643 | 227 | 31071 | 1619 | 1315 | 4065 |
| Consensus349 | 0 | 0 | 0 | 0 | 0 | 0 | 1 | 3 | 21 | 2 | 2 | 1 | 1 | 0 | 1 | 4 | 0 | 4 | 0 |
| Consensus199 | 0 | 0 | 0 | 0 | 0 | 0 | 49 | 1 | 1 | 20 | 4 | 1 | 0 | 18 | 5 | 1 | 19 | 2 | 51 |
| Consensus161 | 0 | 0 | 0 | 0 | 0 | 0 | 0 | 0 | 0 | 0 | 0 | 50 | 0 | 0 | 0 | 0 | 0 | 0 | 211 |
| Consensus7 | 1 | 15 | 6 | 6 | 4 | 5 | 23751 | 4563 | 13239 | 24827 | 5301 | 1025 | 3013 | 4625 | 221 | 31066 | 1600 | 1309 | 3803 |
| Erythrobacteraceae | 0 | 0 | 0 | 0 | 0 | 0 | 0 | 0 | 0 | 0 | 0 | 0 | 0 | 44 | 0 | 0 | 4 | 0 | 0 |
| Consensus328 | 0 | 0 | 0 | 0 | 0 | 0 | 0 | 0 | 0 | 0 | 0 | 0 | 0 | 44 | 0 | 0 | 4 | 0 | 0 |
| Pedobacter | 0 | 0 | 0 | 1 | 0 | 88 | 0 | 4 | 1 | 0 | 0 | 0 | 0 | 112 | 6 | 6 | 138 | 66 | 0 |
| Consensus323 | 0 | 0 | 0 | 0 | 0 | 0 | 0 | 0 | 0 | 0 | 0 | 0 | 0 | 15 | 0 | 0 | 0 | 35 | 0 |
| Consensus282 | 0 | 0 | 0 | 1 | 0 | 74 | 0 | 3 | 1 | 0 | 0 | 0 | 0 | 2 | 0 | 0 | 0 | 0 | 0 |
| Consensus270 | 0 | 0 | 0 | 0 | 0 | 3 | 0 | 1 | 0 | 0 | 0 | 0 | 0 | 22 | 2 | 2 | 59 | 0 | 0 |
| Consensus262 | 0 | 0 | 0 | 0 | 0 | 11 | 0 | 0 | 0 | 0 | 0 | 0 | 0 | 7 | 2 | 2 | 66 | 4 | 0 |
| Consensus239 | 0 | 0 | 0 | 0 | 0 | 0 | 0 | 0 | 0 | 0 | 0 | 0 | 0 | 66 | 2 | 2 | 13 | 27 | 0 |
| Sphingobacterium | 0 | 0 | 0 | 0 | 0 | 9 | 211 | 877 | 6 | 406 | 13 | 115 | 24 | 5759 | 1456 | 6690 | 1793 | 1200 | 2204 |
| Consensus422 | 0 | 0 | 0 | 0 | 0 | 0 | 0 | 0 | 0 | 0 | 1 | 0 | 0 | 4 | 1 | 0 | 1 | 5 | 1 |
| Consensus137 | 0 | 0 | 0 | 0 | 0 | 9 | 0 | 114 | 0 | 2 | 12 | 108 | 24 | 8 | 35 | 2 | 80 | 13 | 0 |
| Consensus136 | 0 | 0 | 0 | 0 | 0 | 0 | 1 | 70 | 0 | 0 | 0 | 0 | 0 | 348 | 0 | 0 | 0 | 0 | 0 |
| Consensus100 | 0 | 0 | 0 | 0 | 0 | 0 | 0 | 0 | 0 | 0 | 0 | 0 | 0 | 1 | 0 | 736 | 6 | 9 | 0 |
| Consensus95 | 0 | 0 | 0 | 0 | 0 | 0 | 0 | 0 | 0 | 0 | 0 | 0 | 0 | 518 | 0 | 2 | 282 | 0 | 0 |
| Consensus81 | 0 | 0 | 0 | 0 | 0 | 0 | 0 | 0 | 0 | 0 | 0 | 0 | 0 | 805 | 0 | 4 | 446 | 2 | 0 |
| Consensus48 | 0 | 0 | 0 | 0 | 0 | 0 | 0 | 0 | 1 | 1 | 0 | 0 | 0 | 2 | 0 | 3549 | 15 | 33 | 1 |
| Consensus39 | 0 | 0 | 0 | 0 | 0 | 0 | 0 | 686 | 5 | 2 | 0 | 5 | 0 | 2239 | 5 | 2113 | 321 | 45 | 5 |
| Consensus32 | 0 | 0 | 0 | 0 | 0 | 0 | 210 | 7 | 0 | 401 | 0 | 2 | 0 | 1834 | 1415 | 284 | 642 | 1093 | 2197 |
| Acinetobacter | 3 | 0 | 2 | 1 | 0 | 24 | 80678 | 5849 | 243 | 1E+05 | 11827 | 45459 | 3693 | 46464 | 5434 | 215 | 28944 | 12021 | 880 |
| Consensus388 | 0 | 0 | 0 | 1 | 0 | 0 | 0 | 0 | 0 | 0 | 0 | 2 | 0 | 0 | 23 | 0 | 0 | 0 | 0 |
| Consensus4 | 3 | 0 | 2 | 0 | 0 | 24 | 80678 | 5849 | 243 | 1E+05 | 11827 | 45457 | 3693 | 46464 | 5411 | 215 | 28944 | 12021 | 880 |
| Desemzia | 0 | 0 | 0 | 0 | 0 | 0 | 0 | 0 | 0 | 0 | 0 | 0 | 0 | 0 | 0 | 0 | 0 | 39 | 0 |
| Consensus351 | 0 | 0 | 0 | 0 | 0 | 0 | 0 | 0 | 0 | 0 | 0 | 0 | 0 | 0 | 0 | 0 | 0 | 39 | 0 |
| Janthinobacterium | 0 | 0 | 0 | 0 | 0 | 0 | 0 | 197 | 0 | 0 | 5 | 0 | 0 | 1 | 0 | 1 | 31 | 2 | 15 |
| Consensus164 | 0 | 0 | 0 | 0 | 0 | 0 | 0 | 197 | 0 | 0 | 5 | 0 | 0 | 1 | 0 | 1 | 31 | 2 | 15 |
| Erwinia | 0 | 0 | 0 | 0 | 0 | 0 | 2 | 1 | 0 | 4 | 3 | 17 | 7 | 3 | 0 | 41 | 0 | 5 | 19 |
| Consensus247 | 0 | 0 | 0 | 0 | 0 | 0 | 2 | 1 | 0 | 4 | 3 | 17 | 7 | 3 | 0 | 41 | 0 | 5 | 19 |
| Sphingomonadaceae | 0 | 0 | 1 | 0 | 0 | 0 | 0 | 0 | 0 | 0 | 0 | 0 | 0 | 0 | 0 | 0 | 29 | 0 | 0 |
| Consensus377 | 0 | 0 | 1 | 0 | 0 | 0 | 0 | 0 | 0 | 0 | 0 | 0 | 0 | 0 | 0 | 0 | 29 | 0 | 0 |
| Selenomonadales | 0 | 0 | 0 | 0 | 0 | 0 | 0 | 0 | 0 | 0 | 0 | 0 | 0 | 0 | 1986 | 15 | 1 | 0 | 0 |
| Consensus291 | 0 | 0 | 0 | 0 | 0 | 0 | 0 | 0 | 0 | 0 | 0 | 0 | 0 | 0 | 70 | 0 | 0 | 0 | 0 |
| Consensus162 | 0 | 0 | 0 | 0 | 0 | 0 | 0 | 0 | 0 | 0 | 0 | 0 | 0 | 0 | 260 | 0 | 0 | 0 | 0 |
| Consensus74 | 0 | 0 | 0 | 0 | 0 | 0 | 0 | 0 | 0 | 0 | 0 | 0 | 0 | 0 | 1656 | 15 | 1 | 0 | 0 |
| Olivibacter | 0 | 0 | 2 | 0 | 0 | 0 | 0 | 0 | 1 | 0 | 0 | 3 | 0 | 198 | 0 | 0 | 65 | 0 | 0 |
| Consensus158 | 0 | 0 | 2 | 0 | 0 | 0 | 0 | 0 | 1 | 0 | 0 | 3 | 0 | 198 | 0 | 0 | 65 | 0 | 0 |
| Lelliottia | 0 | 0 | 0 | 0 | 0 | 0 | 98 | 238 | 241 | 66 | 54 | 107 | 4 | 96 | 46 | 65 | 15 | 371 | 93 |
| Consensus330 | 0 | 0 | 0 | 0 | 0 | 0 | 4 | 0 | 0 | 9 | 2 | 6 | 0 | 8 | 1 | 1 | 5 | 1 | 10 |
| Consensus78 | 0 | 0 | 0 | 0 | 0 | 0 | 94 | 238 | 241 | 57 | 52 | 101 | 4 | 88 | 45 | 64 | 10 | 370 | 83 |
| Kluyvera | 0 | 0 | 0 | 0 | 0 | 0 | 0 | 0 | 0 | 0 | 0 | 878 | 1 | 1 | 1 | 1 | 1 | 0 | 2927 |
| Consensus44 | 0 | 0 | 0 | 0 | 0 | 0 | 0 | 0 | 0 | 0 | 0 | 878 | 1 | 1 | 1 | 1 | 1 | 0 | 2927 |
| Proteobacteria | 0 | 0 | 2 | 3 | 0 | 8 | 2 | 30 | 13 | 0 | 0 | 28 | 0 | 30 | 6 | 1 | 90 | 45 | 64 |
| Consensus150 | 0 | 0 | 2 | 3 | 0 | 8 | 2 | 30 | 13 | 0 | 0 | 28 | 0 | 30 | 6 | 1 | 90 | 45 | 64 |
| Sphingopyxis | 0 | 0 | 6 | 1 | 0 | 3 | 1 | 4 | 0 | 1 | 0 | 0 | 0 | 829 | 51 | 5 | 129 | 489 | 70 |
| Consensus76 | 0 | 0 | 6 | 1 | 0 | 3 | 1 | 4 | 0 | 1 | 0 | 0 | 0 | 829 | 51 | 5 | 129 | 489 | 70 |
| Pseudomonas | 24 | 3 | 18 | 41 | 4 | 325 | 52562 | 3E+05 | 3E+05 | 78641 | 1E+05 | 1E+05 | 47909 | 1E+05 | 17898 | 1E+05 | 5685 | 2E+05 | 47778 |
| Consensus398 | 0 | 0 | 0 | 0 | 0 | 0 | 0 | 0 | 0 | 0 | 0 | 0 | 0 | 0 | 6 | 9 | 0 | 7 | 0 |
| Consensus308 | 0 | 0 | 0 | 0 | 0 | 0 | 3 | 19 | 5 | 2 | 1 | 4 | 0 | 12 | 1 | 10 | 0 | 2 | 0 |
| Consensus307 | 0 | 0 | 0 | 0 | 0 | 0 | 0 | 2 | 0 | 0 | 1 | 0 | 0 | 0 | 0 | 0 | 0 | 0 | 57 |
| Consensus303 | 0 | 0 | 0 | 0 | 0 | 0 | 3 | 11 | 15 | 2 | 8 | 4 | 3 | 0 | 0 | 9 | 0 | 6 | 2 |
| Consensus300 | 0 | 0 | 1 | 0 | 0 | 0 | 6 | 6 | 7 | 4 | 7 | 11 | 0 | 1 | 1 | 0 | 0 | 18 | 2 |
| Consensus280 | 0 | 0 | 0 | 0 | 0 | 1 | 19 | 36 | 0 | 27 | 0 | 0 | 0 | 0 | 0 | 0 | 0 | 0 | 0 |
| Consensus269 | 0 | 0 | 0 | 0 | 0 | 0 | 1 | 18 | 25 | 0 | 9 | 5 | 0 | 17 | 0 | 0 | 0 | 14 | 0 |
| Consensus246 | 0 | 0 | 0 | 0 | 0 | 0 | 6 | 24 | 8 | 12 | 1 | 1 | 0 | 29 | 0 | 20 | 2 | 0 | 0 |

| | | | | | | | | | | | | | | | | | | | |
|---|---|---|---|---|---|---|---|---|---|---|---|---|---|---|---|---|---|---|---|
| Consensus190 | 0 | 0 | 0 | 0 | 0 | 5 | 79 | 21 | 3 | 63 | 2 | 3 | 3 | 0 | 0 | 4 | 5 | 0 | 0 |
| Consensus172 | 0 | 0 | 0 | 0 | 0 | 0 | 7 | 8 | 9 | 2 | 2 | 26 | 0 | 33 | 6 | 81 | 0 | 52 | 1 |
| Consensus145 | 0 | 0 | 1 | 0 | 0 | 0 | 1 | 120 | 33 | 0 | 1 | 0 | 0 | 2 | 0 | 155 | 24 | 1 | 28 |
| Consensus111 | 0 | 0 | 0 | 0 | 0 | 1 | 274 | 46 | 38 | 293 | 0 | 32 | 0 | 0 | 5 | 0 | 1 | 5 | 0 |
| Consensus109 | 0 | 0 | 0 | 0 | 0 | 0 | 235 | 75 | 13 | 202 | 19 | 16 | 2 | 79 | 3 | 23 | 33 | 2 | 1 |
| Consensus63 | 0 | 0 | 0 | 0 | 0 | 20 | 728 | 61 | 14 | 883 | 54 | 46 | 210 | 6 | 11 | 4 | 2 | 7 | 3 |
| Consensus50 | 0 | 0 | 0 | 0 | 0 | 0 | 145 | 658 | 925 | 172 | 748 | 538 | 9 | 70 | 42 | 17 | 1 | 162 | 38 |
| Consensus19 | 0 | 0 | 0 | 0 | 0 | 0 | 884 | 5323 | 4145 | 929 | 3026 | 3082 | 42 | 558 | 752 | 374 | 30 | 3651 | 603 |
| Consensus12 | 1 | 1 | 2 | 0 | 0 | 146 | 7782 | 1969 | 264 | 9474 | 5809 | 4329 | 22665 | 77 | 285 | 116 | 47 | 1319 | 356 |
| Consensus9 | 0 | 0 | 2 | 3 | 0 | 37 | 15722 | 5931 | 1567 | 17535 | 1968 | 861 | 1578 | 21836 | 124 | 4515 | 3766 | 461 | 159 |
| Consensus1 | 23 | 2 | 12 | 38 | 4 | 115 | 26667 | 3E+05 | 3E+05 | 49041 | 1E+05 | 1E+05 | 23397 | 79915 | 16662 | 1E+05 | 1774 | 2E+05 | 46528 |
| Raoultella | 2 | 0 | 0 | 0 | 0 | 0 | 89 | 49 | 13 | 84 | 33 | 54 | 32 | 45 | 763 | 66 | 129 | 312 | 25 |
| Consensus72 | 2 | 0 | 0 | 0 | 0 | 0 | 89 | 49 | 13 | 84 | 33 | 54 | 32 | 45 | 763 | 66 | 129 | 312 | 25 |
| Acidovorax | 0 | 0 | 2 | 1 | 0 | 222 | 0 | 176 | 0 | 0 | 50 | 1 | 0 | 116 | 455 | 16 | 613 | 39 | 203 |
| Consensus147 | 0 | 0 | 1 | 1 | 0 | 221 | 0 | 2 | 0 | 0 | 0 | 0 | 0 | 6 | 0 | 0 | 106 | 5 | 0 |
| Consensus107 | 0 | 0 | 0 | 0 | 0 | 0 | 0 | 0 | 0 | 0 | 0 | 0 | 0 | 105 | 62 | 5 | 472 | 34 | 28 |
| Consensus93 | 0 | 0 | 1 | 0 | 0 | 1 | 0 | 174 | 0 | 0 | 50 | 1 | 0 | 5 | 393 | 11 | 35 | 0 | 175 |
| Veillonellaceae | 0 | 0 | 1 | 0 | 0 | 0 | F | 0 | 0 | 0 | 0 | 0 | 0 | 0 | 8554 | 95 | 1 | 0 | 0 |
| Consensus428 | 0 | 0 | 0 | 0 | 0 | 0 | 0 | 0 | 0 | 0 | 0 | 0 | 0 | 0 | 9 | 0 | 0 | 0 | 0 |
| Consensus369 | 0 | 0 | 0 | 0 | 0 | 0 | 0 | 0 | 0 | 0 | 0 | 0 | 0 | 0 | 33 | 0 | 0 | 0 | 0 |
| Consensus320 | 0 | 0 | 0 | 0 | 0 | 0 | 0 | 0 | 0 | 0 | 0 | 0 | 0 | 0 | 53 | 0 | 0 | 0 | 0 |
| Consensus202 | 0 | 0 | 0 | 0 | 0 | 0 | 0 | 0 | 0 | 0 | 0 | 0 | 0 | 0 | 171 | 0 | 0 | 0 | 0 |
| Consensus120 | 0 | 0 | 0 | 0 | 0 | 0 | 0 | 0 | 0 | 0 | 0 | 0 | 0 | 0 | 547 | 0 | 0 | 0 | 0 |
| Consensus33 | 0 | 0 | 1 | 0 | 0 | 0 | 0 | 0 | 0 | 0 | 0 | 0 | 0 | 0 | 7741 | 95 | 1 | 0 | 0 |
| Paenibacillus | 1 | 0 | 0 | 0 | 0 | 6 | 85 | 1329 | 19 | 91 | 206 | 178 | 35 | 597 | 1565 | 372 | 64 | 1765 | 5321 |
| Consensus427 | 0 | 0 | 0 | 0 | 0 | 0 | 0 | 0 | 0 | 0 | 0 | 0 | 0 | 0 | 0 | 0 | 0 | 4 | 5 |
| Consensus404 | 0 | 0 | 0 | 0 | 0 | 0 | 0 | 0 | 0 | 0 | 0 | 0 | 0 | 0 | 0 | 0 | 0 | 0 | 21 |
| Consensus357 | 0 | 0 | 0 | 0 | 0 | 0 | 1 | 9 | 0 | 0 | 2 | 2 | 0 | 2 | 0 | 3 | 11 | 6 | 0 |
| Consensus295 | 0 | 0 | 0 | 0 | 0 | 0 | 0 | 0 | 0 | 0 | 0 | 0 | 0 | 0 | 6 | 0 | 0 | 2 | 59 |
| Consensus271 | 0 | 0 | 0 | 0 | 0 | 0 | 0 | 0 | 0 | 0 | 0 | 0 | 0 | 0 | 0 | 0 | 0 | 88 | 0 |
| Consensus209 | 0 | 0 | 0 | 0 | 0 | 0 | 0 | 0 | 0 | 0 | 0 | 0 | 0 | 4 | 0 | 0 | 0 | 0 | 153 |
| Consensus176 | 0 | 0 | 0 | 0 | 0 | 0 | 0 | 0 | 0 | 0 | 0 | 0 | 0 | 216 | 0 | 2 | 0 | 0 | 0 |
| Consensus143 | 0 | 0 | 0 | 0 | 0 | 0 | 0 | 7 | 0 | 0 | 0 | 0 | 0 | 0 | 2 | 0 | 0 | 0 | 367 |
| Consensus119 | 0 | 0 | 0 | 0 | 0 | 0 | 0 | 0 | 0 | 0 | 0 | 0 | 0 | 0 | 167 | 3 | 1 | 52 | 337 |
| Consensus117 | 0 | 0 | 0 | 0 | 0 | 0 | 0 | 0 | 0 | 0 | 1 | 39 | 3 | 108 | 60 | 78 | 12 | 299 | 48 |
| Consensus88 | 0 | 0 | 0 | 0 | 0 | 1 | 24 | 538 | 9 | 40 | 86 | 34 | 14 | 70 | 28 | 42 | 11 | 28 | 4 |
| Consensus75 | 0 | 0 | 0 | 0 | 0 | 5 | 60 | 686 | 10 | 51 | 114 | 103 | 18 | 194 | 132 | 121 | 29 | 92 | 30 |
| Consensus34 | 1 | 0 | 0 | 0 | 0 | 0 | 0 | 89 | 0 | 0 | 3 | 0 | 0 | 3 | 1170 | 123 | 0 | 1194 | 4297 |
| Microbacteriaceae | 0 | 0 | 0 | 0 | 0 | 0 | 0 | 0 | 0 | 0 | 0 | 0 | 0 | 0 | 3 | 0 | 9 | 3 | 0 |
| Consensus416 | 0 | 0 | 0 | 0 | 0 | 0 | 0 | 0 | 0 | 0 | 0 | 0 | 0 | 0 | 3 | 0 | 9 | 3 | 0 |
| Brevundimonas | 0 | 0 | 0 | 0 | 0 | 4 | 0 | 428 | 75 | 0 | 0 | 19 | 0 | 359 | 228 | 4 | 757 | 318 | 801 |
| Consensus55 | 0 | 0 | 0 | 0 | 0 | 4 | 0 | 428 | 75 | 0 | 0 | 19 | 0 | 359 | 228 | 4 | 757 | 318 | 801 |
| Rhodobacteraceae | 0 | 0 | 0 | 0 | 0 | 0 | 0 | 0 | 0 | 0 | 0 | 0 | 0 | 12 | 65 | 0 | 1 | 5 | 180 |
| Consensus160 | 0 | 0 | 0 | 0 | 0 | 0 | 0 | 0 | 0 | 0 | 0 | 0 | 0 | 12 | 65 | 0 | 1 | 5 | 180 |
| Oxalicibacterium | 0 | 0 | 3 | 0 | 0 | 0 | 0 | 6 | 18 | 0 | 0 | 18 | 2 | 39 | 6 | 0 | 10 | 32 | 121 |
| Consensus163 | 0 | 0 | 3 | 0 | 0 | 0 | 0 | 6 | 18 | 0 | 0 | 18 | 2 | 39 | 6 | 0 | 10 | 32 | 121 |
| Leucobacter | 0 | 0 | 0 | 0 | 0 | 0 | 0 | 0 | 0 | 0 | 0 | 0 | 0 | 14 | 0 | 0 | 3 | 33 | 0 |
| Consensus324 | 0 | 0 | 0 | 0 | 0 | 0 | 0 | 0 | 0 | 0 | 0 | 0 | 0 | 14 | 0 | 0 | 3 | 33 | 0 |
| Chryseobacterium | 2 | 0 | 0 | 3 | 0 | 21 | 3130 | 8989 | 313 | 3993 | 3609 | 2939 | 628 | 20938 | 1100 | 3866 | 20185 | 6868 | 13823 |
| Consensus337 | 0 | 0 | 0 | 0 | 0 | 0 | 0 | 0 | 0 | 0 | 0 | 0 | 0 | 6 | 0 | 1 | 37 | 0 | 0 |
| Consensus302 | 0 | 0 | 0 | 0 | 0 | 0 | 2 | 30 | 0 | 4 | 2 | 4 | 0 | 1 | 0 | 5 | 13 | 2 | 0 |
| Consensus267 | 0 | 0 | 0 | 0 | 0 | 0 | 8 | 31 | 1 | 4 | 2 | 45 | 0 | 0 | 0 | 0 | 0 | 0 | 0 |
| Consensus258 | 0 | 0 | 0 | 0 | 0 | 2 | 36 | 0 | 0 | 34 | 0 | 0 | 22 | 0 | 0 | 0 | 0 | 0 | 0 |
| Consensus191 | 0 | 0 | 0 | 0 | 0 | 0 | 0 | 0 | 0 | 0 | 0 | 0 | 0 | 87 | 0 | 0 | 0 | 27 | 74 |
| Consensus129 | 0 | 0 | 0 | 0 | 0 | 0 | 5 | 16 | 0 | 1 | 107 | 11 | 0 | 22 | 311 | 2 | 0 | 0 | 0 |
| Consensus128 | 0 | 0 | 0 | 0 | 0 | 0 | 0 | 3 | 0 | 0 | 0 | 0 | 0 | 0 | 0 | 0 | 484 | 0 | 0 |
| Consensus98 | 0 | 0 | 0 | 0 | 0 | 0 | 1 | 0 | 0 | 4 | 0 | 780 | 0 | 0 | 0 | 0 | 2 | 1 | 0 |
| Consensus49 | 0 | 0 | 0 | 0 | 0 | 5 | 103 | 304 | 0 | 114 | 2 | 136 | 0 | 2917 | 1 | 12 | 6 | 0 | 0 |
| Consensus47 | 0 | 0 | 0 | 0 | 0 | 0 | 130 | 219 | 83 | 167 | 2532 | 46 | 7 | 209 | 0 | 0 | 12 | 205 | 0 |
| Consensus22 | 1 | 0 | 0 | 0 | 0 | 0 | 1220 | 128 | 204 | 2052 | 433 | 511 | 24 | 58 | 568 | 598 | 13020 | 79 | 1 |
| Consensus18 | 1 | 0 | 0 | 3 | 0 | 14 | 1625 | 8258 | 25 | 1612 | 528 | 1406 | 575 | 668 | 219 | 3174 | 6603 | 454 | 40 |
| Consensus14 | 0 | 0 | 0 | 0 | 0 | 0 | 0 | 0 | 0 | 1 | 3 | 0 | 0 | 16970 | 1 | 74 | 8 | 6100 | 13708 |
| Novosphingobium | 0 | 0 | 0 | 0 | 0 | 0 | 76 | 332 | 9 | 64 | 2 | 52 | 2 | 17 | 0 | 27 | 132 | 1 | 3 |
| Consensus103 | 0 | 0 | 0 | 0 | 0 | 0 | 76 | 332 | 9 | 64 | 2 | 52 | 2 | 17 | 0 | 27 | 132 | 1 | 3 |
| Methylophilus | 0 | 0 | 0 | 0 | 0 | 0 | 0 | 3 | 0 | 1 | 0 | 0 | 0 | 22 | 0 | 0 | 766 | 0 | 0 |
| Consensus96 | 0 | 0 | 0 | 0 | 0 | 0 | 0 | 3 | 0 | 1 | 0 | 0 | 0 | 22 | 0 | 0 | 766 | 0 | 0 |
| Lachnospiraceae | 0 | 0 | 0 | 0 | 0 | 0 | 0 | 0 | 0 | 0 | 0 | 0 | 0 | 54 | 0 | 394 | 125 | 116 | 17 |
| Consensus106 | 0 | 0 | 0 | 0 | 0 | 0 | 0 | 0 | 0 | 0 | 0 | 0 | 0 | 54 | 0 | 394 | 125 | 116 | 17 |
| Flavobacterium | 0 | 0 | 3 | 0 | 1 | 102 | 12 | 3 | 4 | 17 | 15 | 4595 | 0 | 1715 | 14 | 14 | 3419 | 8602 | 2884 |
| Consensus406 | 0 | 0 | 0 | 0 | 0 | 0 | 0 | 2 | 0 | 0 | 0 | 2 | 0 | 5 | 0 | 0 | 4 | 0 | 8 |
| Consensus360 | 0 | 0 | 0 | 0 | 0 | 0 | 0 | 0 | 0 | 0 | 0 | 33 | 0 | 2 | 0 | 0 | 0 | 0 | 0 |
| Consensus359 | 0 | 0 | 0 | 0 | 0 | 0 | 11 | 0 | 0 | 17 | 0 | 0 | 0 | 7 | 0 | 0 | 0 | 0 | 0 |
| Consensus355 | 0 | 0 | 0 | 0 | 1 | 36 | 0 | 0 | 0 | 0 | 0 | 0 | 0 | 0 | 0 | 0 | 0 | 0 | 0 |
| Consensus339 | 0 | 0 | 0 | 0 | 0 | 13 | 0 | 0 | 0 | 0 | 0 | 0 | 0 | 0 | 0 | 1 | 28 | 1 | 0 |

| | | | | | | | | | | | | | | | | | | | |
|---|---|---|---|---|---|---|---|---|---|---|---|---|---|---|---|---|---|---|---|
| Consensus298 | 0 | 0 | 0 | 0 | 0 | 0 | 0 | 0 | 0 | 0 | 0 | 0 | 0 | 0 | 0 | 0 | 65 | 0 | 0 |
| Consensus287 | 0 | 0 | 3 | 0 | 0 | 53 | 0 | 0 | 0 | 0 | 0 | 2 | 0 | 13 | 0 | 0 | 3 | 1 | 0 |
| Consensus249 | 0 | 0 | 0 | 0 | 0 | 0 | 0 | 0 | 0 | 0 | 0 | 6 | 0 | 32 | 1 | 1 | 42 | 12 | 7 |
| Consensus201 | 0 | 0 | 0 | 0 | 0 | 0 | 0 | 0 | 0 | 0 | 0 | 0 | 0 | 20 | 1 | 0 | 117 | 4 | 29 |
| Consensus183 | 0 | 0 | 0 | 0 | 0 | 0 | 0 | 0 | 0 | 0 | 0 | 0 | 0 | 14 | 0 | 0 | 77 | 80 | 30 |
| Consensus180 | 0 | 0 | 0 | 0 | 0 | 0 | 0 | 0 | 0 | 0 | 0 | 0 | 0 | 0 | 0 | 0 | 9 | 197 | 0 |
| Consensus153 | 0 | 0 | 0 | 0 | 0 | 0 | 0 | 0 | 0 | 0 | 0 | 1 | 0 | 255 | 1 | 3 | 0 | 5 | 32 |
| Consensus69 | 0 | 0 | 0 | 0 | 0 | 0 | 1 | 0 | 4 | 0 | 1 | 1758 | 0 | 0 | 2 | 0 | 13 | 1 | 3 |
| Consensus53 | 0 | 0 | 0 | 0 | 0 | 0 | 0 | 1 | 0 | 0 | 0 | 2793 | 0 | 311 | 0 | 4 | 25 | 38 | 11 |
| Consensus24 | 0 | 0 | 0 | 0 | 0 | 0 | 0 | 0 | 0 | 0 | 14 | 0 | 0 | 1056 | 9 | 5 | 3036 | 8263 | 2764 |
| Arthrobacter | 0 | 0 | 1 | 0 | 0 | 0 | 56 | 0 | 0 | 80 | 0 | 0 | 0 | 64 | 0 | 0 | 220 | 18 | 13 |
| Consensus131 | 0 | 0 | 1 | 0 | 0 | 0 | 56 | 0 | 0 | 80 | 0 | 0 | 0 | 64 | 0 | 0 | 220 | 18 | 13 |
| Ochrobactrum | 0 | 0 | 0 | 0 | 0 | 0 | 156 | 12 | 32 | 84 | 32 | 70 | 3 | 11576 | 960 | 3051 | 15946 | 2729 | 1060 |
| Consensus420 | 0 | 0 | 0 | 0 | 0 | 0 | 0 | 0 | 0 | 0 | 0 | 0 | 0 | 3 | 2 | 0 | 2 | 7 | 0 |
| Consensus331 | 0 | 0 | 0 | 0 | 0 | 0 | 0 | 0 | 0 | 0 | 0 | 1 | 0 | 9 | 13 | 0 | 14 | 7 | 2 |
| Consensus297 | 0 | 0 | 0 | 0 | 0 | 0 | 0 | 0 | 0 | 0 | 0 | 0 | 0 | 20 | 3 | 6 | 33 | 3 | 0 |
| Consensus90 | 0 | 0 | 0 | 0 | 0 | 0 | 3 | 0 | 0 | 0 | 0 | 5 | 0 | 222 | 17 | 82 | 445 | 79 | 17 |
| Consensus87 | 0 | 0 | 0 | 0 | 0 | 0 | 53 | 5 | 7 | 38 | 0 | 4 | 0 | 415 | 122 | 2 | 19 | 128 | 162 |
| Consensus28 | 0 | 0 | 0 | 0 | 0 | 0 | 56 | 7 | 21 | 27 | 29 | 22 | 1 | 5699 | 700 | 36 | 3249 | 1240 | 686 |
| Consensus20 | 0 | 0 | 0 | 0 | 0 | 0 | 44 | 0 | 4 | 19 | 3 | 38 | 2 | 5208 | 103 | 2925 | 12184 | 1265 | 193 |
| Flavobacteriaceae | 0 | 0 | 0 | 0 | 0 | 0 | 7 | 0 | 0 | 3 | 0 | 13 | 0 | 9 | 9 | 2 | 8 | 2 | 1 |
| Consensus315 | 0 | 0 | 0 | 0 | 0 | 0 | 7 | 0 | 0 | 3 | 0 | 13 | 0 | 9 | 9 | 2 | 8 | 2 | 1 |

B

| OTU | **K1m** | **K2m** | **K4m** | **K5m** | **K6m** | **2d1m** | **2d2m** | **2d3m** | **2d4m** | **2d5m** | **5d1m** | **5d2m** | **5d3m** | **5d4m** | **5d5m** | **8d3m** | **8d4m** | **8d5m** | **8d6m** | **8d7m** |
|---|---|---|---|---|---|---|---|---|---|---|---|---|---|---|---|---|---|---|---|---|
| Consensus1 | 0 | 0 | 0 | 0 | 0 | 6 | 3 | 0 | 16 | 7 | 0 | 56 | 9 | 10 | 585 | 7101 | 0 | 2 | 0 | 2E+05 |
| Consensus2 | 0 | 4 | 3 | 2 | 3 | 2 | 0 | 2 | 0 | 0 | 5 | 6 | 129 | 98 | 1279 | 312 | 5 | 859 | 41 | 9 |
| Consensus3 | 0 | 0 | 0 | 0 | 0 | 0 | 2 | 0 | 0 | 0 | 0 | 1 | 8 | 0 | 0 | 0 | 0 | 0 | 0 | 2 |
| Consensus4 | 0 | 0 | 0 | 0 | 0 | 1 | 0 | 0 | 0 | 0 | 0 | 12 | 0 | 0 | 3 | 2 | 0 | 0 | 3 | 2 |
| Consensus6 | 0 | 0 | 0 | 0 | 0 | 0 | 1 | 0 | 0 | 2 | 0 | 1 | 15 | 2 | 8 | 65 | 1 | 15 | 0 | 11764 |
| Consensus7 | 0 | 0 | 0 | 0 | 0 | 0 | 10 | 0 | 3 | 2 | 4 | 8 | 7 | 38 | 17 | 3479 | 5 | 47 | 20 | 8 |
| Consensus9 | 0 | 1 | 0 | 0 | 0 | 25 | 123 | 170 | 14 | 195 | 2 | 9 | 41 | 3347 | 3526 | 3 | 0 | 27 | 7 | 2763 |
| Consensus10 | 0 | 0 | 0 | 10 | 0 | 0 | 9 | 1 | 1 | 5 | 0 | 3 | 2 | 3 | 6 | 113 | 0 | 6 | 0 | 12505 |
| Consensus11 | 0 | 2 | 5 | 0 | 2 | 2 | 0 | 3 | 0 | 0 | 0 | 0 | 2 | 3 | 5 | 10336 | 2 | 3 | 69 | 0 |
| Consensus12 | 6 | 0 | 0 | 0 | 0 | 42 | 2 | 42 | 1 | 0 | 6 | 20 | 152 | 168 | 3084 | 234 | 1 | 4 | 5 | 906 |
| Consensus13 | 0 | 0 | 0 | 0 | 0 | 0 | 0 | 0 | 0 | 0 | 0 | 0 | 0 | 0 | 3 | 0 | 0 | 0 | 0 | 21 |
| Consensus14 | 0 | 0 | 0 | 0 | 0 | 0 | 0 | 0 | 0 | 0 | 0 | 0 | 0 | 0 | 1 | 0 | 0 | 0 | 0 | 0 |
| Consensus15 | 0 | 0 | 0 | 0 | 0 | 2 | 0 | 1 | 1 | 0 | 2 | 0 | 0 | 1 | 3 | 1 | 4 | 10 | 1 | 0 |
| Consensus16 | 0 | 0 | 2 | 2 | 0 | 1 | 0 | 0 | 0 | 4 | 0 | 3 | 430 | 16 | 126 | 263 | 6 | 412 | 0 | 267 |
| Consensus17 | 1 | 3 | 0 | 1 | 0 | 59 | 0 | 26 | 26 | 0 | 48 | 0 | 12 | 34 | 102 | 42183 | 79 | 147 | 43 | 0 |
| Consensus18 | 0 | 0 | 0 | 0 | 0 | 0 | 0 | 0 | 0 | 0 | 0 | 0 | 0 | 0 | 0 | 44 | 0 | 0 | 0 | 0 |
| Consensus19 | 0 | 0 | 0 | 0 | 0 | 1 | 0 | 0 | 1 | 1 | 0 | 1 | 4 | 29 | 15 | 22 | 0 | 0 | 0 | 675 |
| Consensus20 | 0 | 0 | 0 | 0 | 0 | 0 | 0 | 0 | 0 | 0 | 0 | 0 | 0 | 0 | 0 | 0 | 0 | 0 | 0 | 5 |
| Consensus21 | 0 | 1 | 0 | 2 | 1 | 3 | 0 | 0 | 0 | 2 | 13 | 1 | 116 | 7 | 1436 | 29 | 0 | 21 | 0 | 12623 |
| Consensus22 | 0 | 0 | 0 | 0 | 0 | 0 | 0 | 0 | 0 | 0 | 0 | 0 | 0 | 0 | 0 | 0 | 0 | 0 | 2 | 0 |
| Consensus23 | 0 | 0 | 0 | 0 | 0 | 0 | 0 | 0 | 0 | 0 | 0 | 0 | 0 | 0 | 81 | 6 | 0 | 0 | 0 | 0 |
| Consensus25 | 0 | 0 | 0 | 0 | 0 | 2 | 88 | 0 | 20 | 0 | 0 | 0 | 102 | 7 | 0 | 1 | 0 | 0 | 0 | 5 |
| Consensus26 | 0 | 0 | 0 | 0 | 0 | 57 | 29 | 0 | 0 | 43 | 1 | 1 | 0 | 0 | 0 | 0 | 0 | 0 | 0 | 0 |
| Consensus28 | 0 | 0 | 0 | 0 | 0 | 0 | 0 | 0 | 0 | 0 | 0 | 0 | 0 | 0 | 0 | 0 | 0 | 0 | 0 | 0 |
| Consensus29 | 0 | 8 | 0 | 7 | 3 | 478 | 0 | 227 | 243 | 1 | 528 | 3 | 51 | 686 | 483 | 326 | 550 | 216 | 310 | 664 |
| Consensus33 | 0 | 0 | 0 | 0 | 0 | 0 | 0 | 0 | 0 | 0 | 0 | 0 | 0 | 4 | 0 | 0 | 4 | 0 | 0 | 0 |
| Consensus34 | 0 | 0 | 0 | 0 | 0 | 0 | 0 | 0 | 0 | 0 | 0 | 0 | 0 | 0 | 0 | 0 | 0 | 0 | 0 | 0 |
| Consensus37 | 3 | 10 | 1 | 3 | 4 | 4 | 0 | 5 | 1 | 0 | 1 | 0 | 318 | 316 | 316 | 937 | 15 | 5832 | 69 | 0 |
| Consensus38 | 0 | 0 | 0 | 2 | 0 | 0 | 0 | 0 | 0 | 0 | 1 | 0 | 0 | 0 | 5 | 0 | 0 | 0 | 0 | 587 |
| Consensus39 | 0 | 0 | 0 | 0 | 0 | 0 | 0 | 0 | 0 | 0 | 0 | 0 | 0 | 0 | 0 | 0 | 0 | 0 | 1 | 0 |
| Consensus40 | 0 | 0 | 0 | 0 | 0 | 0 | 0 | 0 | 0 | 0 | 0 | 2 | 0 | 0 | 0 | 0 | 0 | 0 | 0 | 36 |
| Consensus42 | 0 | 0 | 0 | 0 | 0 | 0 | 0 | 0 | 0 | 0 | 0 | 0 | 0 | 0 | 0 | 0 | 0 | 0 | 0 | 0 |
| Consensus44 | 0 | 0 | 0 | 1 | 0 | 1 | 0 | 0 | 0 | 0 | 0 | 0 | 0 | 0 | 2 | 12371 | 0 | 1903 | 0 | 0 |
| Consensus45 | 0 | 0 | 0 | 0 | 0 | 0 | 83 | 0 | 4 | 0 | 0 | 1 | 3 | 0 | 52 | 4 | 0 | 0 | 0 | 8 |
| Consensus46 | 0 | 2 | 2 | 7 | 2 | 817 | 40 | 356 | 376 | 31 | 834 | 66 | 36 | 315 | 800 | 282 | 836 | 302 | 480 | 79 |
| Consensus47 | 0 | 0 | 0 | 0 | 0 | 0 | 0 | 0 | 0 | 0 | 0 | 0 | 0 | 0 | 1 | 0 | 0 | 0 | 0 | 2 |
| Consensus50 | 0 | 0 | 0 | 0 | 0 | 6 | 0 | 0 | 0 | 0 | 0 | 1 | 1 | 2 | 64 | 157 | 0 | 0 | 0 | 189 |
| Consensus52 | 0 | 0 | 0 | 0 | 0 | 0 | 0 | 0 | 0 | 0 | 0 | 0 | 0 | 0 | 0 | 0 | 0 | 0 | 0 | 0 |
| Consensus54 | 0 | 8 | 0 | 1 | 1 | 2 | 110 | 2 | 2 | 0 | 5 | 9 | 109 | 84 | 103 | 98 | 78 | 766 | 171 | 7037 |
| Consensus55 | 0 | 0 | 0 | 1 | 0 | 0 | 0 | 0 | 0 | 0 | 0 | 0 | 0 | 0 | 1 | 0 | 0 | 0 | 0 | 0 |
| Consensus59 | 0 | 0 | 0 | 0 | 0 | 0 | 0 | 1 | 0 | 0 | 0 | 0 | 42 | 4 | 4 | 139 | 16 | 21 | 0 | 69 |
| Consensus63 | 0 | 0 | 0 | 0 | 0 | 2 | 245 | 0 | 0 | 3 | 1 | 4 | 23 | 2 | 2028 | 3 | 0 | 0 | 0 | 738 |
| Consensus65 | 0 | 0 | 0 | 0 | 0 | 0 | 0 | 0 | 0 | 0 | 0 | 0 | 0 | 0 | 0 | 0 | 0 | 0 | 0 | 0 |
| Consensus66 | 0 | 8 | 0 | 0 | 0 | 0 | 0 | 0 | 0 | 3 | 0 | 0 | 42 | 4 | 9 | 23 | 206 | 0 | 0 | 1653 |
| Consensus69 | 0 | 3 | 0 | 0 | 0 | 0 | 0 | 0 | 0 | 0 | 0 | 0 | 0 | 0 | 0 | 0 | 0 | 0 | 0 | 0 |
| Consensus72 | 0 | 0 | 6 | 0 | 0 | 0 | 0 | 4 | 0 | 0 | 0 | 0 | 7 | 0 | 64 | 100 | 0 | 53014 | 0 | 0 |
| Consensus74 | 0 | 1 | 0 | 0 | 0 | 0 | 0 | 0 | 0 | 0 | 0 | 0 | 0 | 2 | 0 | 0 | 0 | 0 | 0 | 0 |

| | | | | | | | | | | | | | | | | | | | | |
|---|---|---|---|---|---|---|---|---|---|---|---|---|---|---|---|---|---|---|---|---|
| Consensus75 | 0 | 0 | 0 | 0 | 0 | 4 | 27 | 0 | 14 | 611 | 182 | 2030 | 10 | 4 | 161 | 5 | 306 | 4 | 0 | 2472 |
| Consensus76 | 0 | 0 | 0 | 1 | 0 | 0 | 0 | 0 | 0 | 0 | 0 | 0 | 0 | 0 | 0 | 0 | 0 | 0 | 0 | 0 |
| Consensus78 | 1 | 4 | 0 | 0 | 0 | 2 | 0 | 0 | 0 | 0 | 0 | 0 | 515 | 372 | 408 | 50 | 6 | 86 | 17 | 42 |
| Consensus81 | 0 | 0 | 0 | 0 | 0 | 1 | 0 | 0 | 0 | 0 | 0 | 0 | 0 | 0 | 0 | 0 | 0 | 0 | 0 | 0 |
| Consensus85 | 0 | 2 | 0 | 0 | 0 | 0 | 0 | 0 | 0 | 1 | 0 | 0 | 18 | 1 | 1 | 17 | 2 | 27 | 0 | 1 |
| Consensus86 | 0 | 9 | 0 | 8 | 0 | 5 | 0 | 1 | 0 | 0 | 2 | 0 | 1 | 0 | 0 | 1 | 0 | 0 | 1 | 5 |
| Consensus87 | 0 | 0 | 0 | 0 | 0 | 0 | 0 | 0 | 0 | 0 | 0 | 0 | 0 | 0 | 0 | 0 | 0 | 0 | 0 | 0 |
| Consensus88 | 0 | 0 | 0 | 0 | 0 | 1 | 14 | 0 | 3 | 158 | 0 | 594 | 4 | 4 | 66 | 0 | 66 | 2 | 0 | 1052 |
| Consensus90 | 0 | 0 | 0 | 0 | 0 | 0 | 0 | 0 | 0 | 0 | 0 | 0 | 0 | 0 | 0 | 0 | 0 | 0 | 0 | 93 |
| Consensus91 | 0 | 2 | 0 | 0 | 1 | 0 | 6 | 0 | 0 | 0 | 0 | 0 | 0 | 0 | 0 | 0 | 0 | 0 | 0 | 1 |
| Consensus98 | 0 | 0 | 0 | 0 | 0 | 0 | 0 | 0 | 0 | 0 | 0 | 0 | 0 | 0 | 0 | 0 | 0 | 0 | 0 | 0 |
| Consensus102 | 0 | 0 | 0 | 0 | 0 | 0 | 0 | 0 | 0 | 0 | 0 | 0 | 0 | 0 | 0 | 0 | 0 | 0 | 0 | 0 |
| Consensus103 | 0 | 0 | 0 | 0 | 0 | 1 | 0 | 0 | 0 | 0 | 0 | 0 | 0 | 0 | 2 | 0 | 0 | 0 | 0 | 0 |
| Consensus104 | 0 | 1 | 0 | 0 | 0 | 0 | 0 | 0 | 0 | 0 | 0 | 0 | 0 | 0 | 0 | 0 | 0 | 0 | 0 | 0 |
| Consensus105 | 0 | 1 | 0 | 0 | 0 | 176 | 225 | 0 | 1 | 616 | 5 | 0 | 0 | 0 | 0 | 0 | 0 | 0 | 0 | 0 |
| Consensus106 | 0 | 0 | 0 | 0 | 0 | 0 | 0 | 0 | 0 | 0 | 0 | 0 | 0 | 0 | 0 | 0 | 0 | 0 | 0 | 0 |
| Consensus109 | 0 | 0 | 0 | 1 | 0 | 0 | 4 | 22 | 0 | 19 | 0 | 0 | 14 | 1256 | 246 | 4 | 0 | 0 | 0 | 49 |
| Consensus111 | 0 | 0 | 0 | 0 | 0 | 0 | 1 | 2 | 0 | 0 | 0 | 1 | 0 | 0 | 18 | 0 | 0 | 0 | 0 | 0 |
| Consensus112 | 0 | 0 | 0 | 0 | 0 | 0 | 0 | 1 | 0 | 0 | 0 | 0 | 48 | 1 | 3 | 0 | 2 | 0 | 0 | 0 |
| Consensus116 | 0 | 0 | 0 | 0 | 0 | 0 | 0 | 0 | 0 | 0 | 0 | 0 | 3 | 0 | 0 | 1 | 0 | 0 | 0 | 58 |
| Consensus117 | 0 | 0 | 0 | 0 | 0 | 0 | 0 | 0 | 0 | 15 | 0 | 0 | 0 | 0 | 6 | 0 | 0 | 0 | 0 | 0 |
| Consensus119 | 0 | 0 | 0 | 0 | 0 | 0 | 0 | 0 | 0 | 0 | 0 | 7030 | 0 | 0 | 3 | 0 | 0 | 0 | 0 | 1 |
| Consensus126 | 0 | 0 | 0 | 0 | 0 | 0 | 0 | 0 | 0 | 0 | 0 | 0 | 0 | 0 | 0 | 0 | 2 | 1 | 0 | 27 |
| Consensus131 | 0 | 0 | 0 | 0 | 0 | 0 | 4 | 0 | 0 | 0 | 0 | 0 | 0 | 0 | 0 | 0 | 0 | 7 | 0 | 3 |
| Consensus134 | 0 | 0 | 0 | 1 | 0 | 0 | 0 | 0 | 0 | 0 | 1 | 1 | 0 | 0 | 0 | 0 | 0 | 0 | 0 | 0 |
| Consensus137 | 0 | 0 | 0 | 0 | 0 | 0 | 0 | 0 | 0 | 0 | 0 | 0 | 0 | 0 | 0 | 0 | 0 | 0 | 0 | 2 |
| Consensus138 | 0 | 107 | 29 | 295 | 21 | 51 | 18 | 19 | 18 | 22 | 54 | 5 | 0 | 3 | 29 | 40 | 0 | 12 | 18 | 8 |
| Consensus140 | 0 | 0 | 0 | 0 | 0 | 0 | 0 | 0 | 0 | 0 | 0 | 0 | 1 | 0 | 4 | 0 | 0 | 0 | 0 | 258 |
| Consensus142 | 0 | 0 | 0 | 0 | 0 | 0 | 0 | 0 | 0 | 0 | 0 | 0 | 0 | 0 | 0 | 0 | 0 | 0 | 0 | 0 |
| Consensus144 | 0 | 0 | 0 | 0 | 0 | 0 | 0 | 0 | 1 | 0 | 0 | 0 | 0 | 0 | 0 | 0 | 0 | 0 | 0 | 0 |
| Consensus145 | 0 | 0 | 0 | 0 | 0 | 0 | 0 | 0 | 0 | 0 | 0 | 256 | 4 | 0 | 1 | 0 | 0 | 0 | 0 | 0 |
| Consensus147 | 0 | 2 | 0 | 0 | 0 | 0 | 0 | 0 | 0 | 0 | 0 | 0 | 0 | 0 | 2 | 0 | 0 | 0 | 0 | 207 |
| Consensus148 | 1 | 0 | 0 | 0 | 0 | 0 | 0 | 0 | 0 | 0 | 0 | 0 | 0 | 0 | 4 | 2 | 0 | 0 | 0 | 0 |
| Consensus150 | 0 | 4 | 0 | 0 | 0 | 0 | 0 | 0 | 0 | 0 | 0 | 0 | 0 | 0 | 0 | 0 | 0 | 0 | 0 | 0 |
| Consensus151 | 0 | 0 | 0 | 0 | 0 | 0 | 0 | 0 | 0 | 0 | 0 | 0 | 1 | 1 | 0 | 0 | 0 | 0 | 0 | 486 |
| Consensus153 | 0 | 0 | 0 | 0 | 0 | 0 | 0 | 0 | 0 | 0 | 0 | 0 | 0 | 0 | 0 | 0 | 0 | 0 | 0 | 0 |
| Consensus154 | 0 | 0 | 0 | 0 | 0 | 0 | 0 | 0 | 0 | 0 | 0 | 0 | 0 | 0 | 0 | 0 | 0 | 0 | 0 | 0 |
| Consensus156 | 0 | 0 | 0 | 0 | 0 | 0 | 0 | 0 | 0 | 0 | 0 | 0 | 0 | 0 | 0 | 0 | 0 | 0 | 0 | 0 |
| Consensus161 | 0 | 0 | 0 | 0 | 0 | 0 | 0 | 0 | 0 | 0 | 0 | 0 | 0 | 1 | 0 | 61 | 0 | 4 | 0 | 0 |
| Consensus162 | 0 | 0 | 0 | 0 | 0 | 0 | 0 | 0 | 0 | 0 | 0 | 0 | 0 | 1 | 0 | 0 | 1 | 0 | 0 | 0 |
| Consensus164 | 0 | 0 | 0 | 0 | 0 | 0 | 0 | 0 | 0 | 0 | 1 | 0 | 0 | 0 | 2 | 0 | 0 | 0 | 0 | 0 |
| Consensus172 | 0 | 0 | 0 | 0 | 0 | 0 | 0 | 0 | 0 | 0 | 0 | 0 | 1 | 0 | 5 | 11 | 0 | 0 | 0 | 30 |
| Consensus174 | 0 | 1 | 0 | 0 | 0 | 0 | 0 | 0 | 0 | 0 | 0 | 0 | 0 | 0 | 0 | 0 | 0 | 0 | 0 | 0 |
| Consensus175 | 2 | 8 | 0 | 9 | 4 | 6 | 0 | 2 | 2 | 0 | 0 | 0 | 178 | 177 | 214 | 100 | 9 | 2054 | 53 | 5 |
| Consensus176 | 0 | 0 | 0 | 0 | 0 | 0 | 0 | 0 | 0 | 0 | 0 | 0 | 0 | 0 | 0 | 0 | 0 | 0 | 0 | 7 |
| Consensus177 | 0 | 0 | 0 | 0 | 0 | 0 | 0 | 0 | 0 | 0 | 0 | 0 | 0 | 0 | 0 | 0 | 0 | 0 | 0 | 0 |
| Consensus181 | 2 | 0 | 0 | 4 | 1 | 0 | 687 | 0 | 1 | 2 | 2 | 0 | 1 | 2 | 4 | 0 | 0 | 1 | 16840 | 885 |
| Consensus185 | 0 | 0 | 0 | 0 | 0 | 0 | 0 | 0 | 0 | 0 | 0 | 0 | 0 | 0 | 0 | 0 | 0 | 0 | 0 | 0 |
| Consensus190 | 1 | 1 | 0 | 0 | 0 | 0 | 16 | 0 | 27 | 0 | 2 | 1 | 0 | 0 | 4 | 0 | 0 | 7 | 0 | 2473 |
| Consensus193 | 0 | 0 | 0 | 0 | 0 | 0 | 1 | 0 | 0 | 0 | 0 | 0 | 0 | 0 | 1 | 0 | 0 | 0 | 0 | 0 |
| Consensus195 | 0 | 0 | 0 | 0 | 0 | 0 | 0 | 0 | 0 | 0 | 0 | 147 | 0 | 0 | 0 | 0 | 0 | 1 | 0 | 29 |
| Consensus196 | 1 | 19 | 1 | 21 | 9 | 15 | 0 | 9 | 8 | 0 | 5 | 0 | 728 | 578 | 735 | 529 | 56 | 5064 | 187 | 31 |
| Consensus197 | 0 | 0 | 0 | 0 | 0 | 0 | 0 | 0 | 0 | 0 | 0 | 0 | 0 | 0 | 0 | 0 | 0 | 0 | 0 | 0 |
| Consensus199 | 0 | 1 | 0 | 0 | 0 | 2 | 25 | 3 | 0 | 4 | 0 | 17 | 24 | 327 | 34 | 2652 | 7 | 128 | 116 | 3635 |
| Consensus209 | 0 | 0 | 0 | 0 | 0 | 0 | 0 | 0 | 0 | 0 | 0 | 0 | 0 | 0 | 0 | 0 | 0 | 0 | 0 | 0 |
| Consensus210 | 0 | 0 | 0 | 0 | 0 | 0 | 0 | 0 | 0 | 0 | 0 | 0 | 0 | 0 | 0 | 0 | 0 | 0 | 0 | 1 |
| Consensus211 | 0 | 0 | 0 | 0 | 0 | 0 | 5 | 0 | 0 | 7 | 0 | 28 | 0 | 0 | 0 | 0 | 0 | 0 | 0 | 7 |
| Consensus213 | 0 | 0 | 0 | 0 | 0 | 0 | 0 | 0 | 0 | 0 | 0 | 0 | 0 | 0 | 0 | 0 | 0 | 0 | 0 | 0 |
| Consensus216 | 0 | 0 | 0 | 0 | 0 | 0 | 0 | 0 | 0 | 0 | 0 | 0 | 0 | 0 | 0 | 0 | 0 | 0 | 0 | 0 |
| Consensus219 | 0 | 0 | 0 | 0 | 0 | 0 | 0 | 0 | 0 | 0 | 0 | 0 | 10 | 6 | 3 | 179 | 0 | 12 | 0 | 28 |
| Consensus221 | 1 | 4 | 0 | 1 | 1 | 3 | 2 | 1 | 2 | 0 | 0 | 5 | 744 | 385 | 783 | 107 | 7 | 108 | 18 | 6217 |
| Consensus223 | 2 | 13 | 0 | 21 | 7 | 43 | 0 | 18 | 18 | 0 | 40 | 1 | 307 | 307 | 91 | 220 | 81 | 603 | 64 | 8 |
| Consensus226 | 1 | 0 | 0 | 0 | 0 | 0 | 0 | 0 | 0 | 0 | 0 | 0 | 0 | 0 | 2 | 0 | 0 | 0 | 0 | 0 |
| Consensus237 | 0 | 0 | 0 | 0 | 0 | 0 | 0 | 0 | 0 | 0 | 0 | 0 | 0 | 0 | 1 | 0 | 0 | 0 | 0 | 0 |
| Consensus239 | 0 | 4 | 0 | 0 | 0 | 0 | 0 | 0 | 0 | 0 | 0 | 0 | 0 | 0 | 4 | 0 | 0 | 0 | 0 | 0 |
| Consensus242 | 0 | 0 | 0 | 0 | 0 | 0 | 0 | 0 | 0 | 0 | 0 | 0 | 0 | 0 | 0 | 2 | 0 | 0 | 0 | 0 |
| Consensus245 | 0 | 0 | 0 | 0 | 0 | 0 | 0 | 0 | 0 | 0 | 0 | 0 | 0 | 0 | 0 | 0 | 1 | 0 | 0 | 78 |
| Consensus246 | 0 | 0 | 0 | 0 | 0 | 0 | 0 | 0 | 10 | 0 | 1 | 0 | 2 | 1 | 212 | 3005 | 0 | 1 | 0 | 33 |
| Consensus247 | 0 | 0 | 0 | 0 | 0 | 0 | 0 | 0 | 0 | 0 | 0 | 0 | 0 | 0 | 0 | 1 | 0 | 565 | 0 | 0 |
| Consensus259 | 0 | 0 | 0 | 0 | 0 | 0 | 0 | 0 | 0 | 0 | 0 | 0 | 0 | 0 | 0 | 0 | 0 | 0 | 0 | 0 |
| Consensus268 | 0 | 0 | 0 | 1 | 0 | 0 | 0 | 0 | 0 | 0 | 0 | 0 | 46 | 7 | 3 | 196 | 0 | 19 | 0 | 6 |
| Consensus269 | 0 | 0 | 0 | 1 | 0 | 38 | 13 | 0 | 0 | 0 | 0 | 62 | 41 | 5 | 51 | 558 | 0 | 0 | 0 | 579 |
| Consensus270 | 0 | 6 | 0 | 0 | 0 | 0 | 0 | 0 | 0 | 0 | 0 | 0 | 0 | 0 | 0 | 2 | 0 | 0 | 0 | 2 |
| Consensus271 | 0 | 0 | 0 | 0 | 0 | 0 | 0 | 0 | 0 | 0 | 0 | 0 | 0 | 0 | 0 | 0 | 0 | 0 | 0 | 0 |

| | | | | | | | | | | | | | | | | | | | | |
|---|---|---|---|---|---|---|---|---|---|---|---|---|---|---|---|---|---|---|---|---|
| Consensus273 | 0 | 0 | 0 | 0 | 0 | 0 | 0 | 0 | 0 | 0 | 0 | 2 | 0 | 0 | 0 | 0 | 0 | 0 | 0 | 16 |
| Consensus279 | 0 | 1 | 0 | 0 | 0 | 0 | 0 | 0 | 0 | 0 | 0 | 0 | 0 | 0 | 0 | 0 | 0 | 0 | 0 | 0 |
| Consensus280 | 0 | 0 | 0 | 0 | 0 | 0 | 49 | 2 | 0 | 0 | 0 | 0 | 0 | 1 | 82 | 0 | 0 | 0 | 0 | 0 |
| Consensus282 | 0 | 5 | 0 | 1 | 0 | 0 | 0 | 0 | 0 | 0 | 0 | 3 | 0 | 0 | 4 | 0 | 0 | 0 | 0 | 0 |
| Consensus284 | 0 | 0 | 0 | 1 | 0 | 2 | 0 | 0 | 5 | 0 | 0 | 0 | 8 | 1 | 0 | 0 | 0 | 0 | 0 | 0 |
| Consensus287 | 0 | 2 | 0 | 0 | 0 | 0 | 0 | 0 | 0 | 0 | 0 | 0 | 0 | 0 | 0 | 0 | 0 | 0 | 0 | 0 |
| Consensus289 | 0 | 0 | 0 | 0 | 0 | 141 | 0 | 67 | 86 | 0 | 163 | 0 | 12 | 65 | 139 | 107 | 162 | 115 | 95 | 0 |
| Consensus293 | 0 | 0 | 0 | 0 | 0 | 1 | 1 | 0 | 0 | 0 | 0 | 0 | 18 | 343 | 21 | 13 | 1 | 0 | 0 | 87 |
| Consensus297 | 0 | 0 | 0 | 0 | 0 | 0 | 0 | 0 | 0 | 0 | 0 | 0 | 0 | 0 | 0 | 0 | 0 | 0 | 0 | 1235 |
| Consensus300 | 0 | 0 | 0 | 0 | 0 | 0 | 0 | 0 | 0 | 0 | 0 | 0 | 0 | 0 | 0 | 0 | 0 | 0 | 0 | 2 |
| Consensus302 | 0 | 0 | 0 | 0 | 0 | 0 | 0 | 0 | 0 | 0 | 0 | 1 | 0 | 0 | 0 | 1 | 0 | 0 | 0 | 0 |
| Consensus303 | 0 | 0 | 0 | 0 | 0 | 0 | 0 | 0 | 0 | 0 | 0 | 0 | 0 | 0 | 0 | 2 | 0 | 0 | 0 | 3 |
| Consensus304 | 0 | 1 | 0 | 0 | 0 | 0 | 0 | 0 | 0 | 0 | 0 | 0 | 7 | 0 | 2 | 1 | 0 | 0 | 0 | 0 |
| Consensus307 | 0 | 0 | 0 | 0 | 0 | 0 | 0 | 0 | 0 | 0 | 0 | 0 | 0 | 0 | 5 | 0 | 0 | 0 | 0 | 0 |
| Consensus308 | 0 | 0 | 0 | 0 | 0 | 0 | 0 | 0 | 0 | 0 | 0 | 0 | 0 | 0 | 3 | 20 | 0 | 0 | 0 | 29 |
| Consensus309 | 0 | 0 | 0 | 0 | 0 | 0 | 0 | 0 | 0 | 0 | 0 | 0 | 0 | 0 | 0 | 0 | 0 | 0 | 0 | 38 |
| Consensus312 | 0 | 0 | 0 | 0 | 0 | 0 | 0 | 0 | 0 | 0 | 1 | 0 | 6 | 75 | 12 | 2 | 5 | 0 | 4 | 46 |
| Consensus315 | 0 | 0 | 0 | 0 | 0 | 0 | 0 | 0 | 0 | 0 | 0 | 0 | 0 | 0 | 0 | 0 | 0 | 0 | 0 | 0 |
| Consensus317 | 0 | 0 | 0 | 0 | 0 | 0 | 0 | 0 | 0 | 0 | 0 | 0 | 0 | 0 | 0 | 22 | 0 | 0 | 0 | 87 |
| Consensus319 | 0 | 0 | 0 | 0 | 0 | 1 | 0 | 0 | 0 | 0 | 0 | 0 | 0 | 0 | 0 | 0 | 0 | 0 | 0 | 0 |
| Consensus321 | 0 | 0 | 0 | 0 | 0 | 0 | 0 | 3 | 0 | 0 | 0 | 0 | 14 | 253 | 12 | 7 | 3 | 0 | 1 | 27 |
| Consensus322 | 0 | 0 | 1 | 0 | 0 | 0 | 0 | 0 | 0 | 0 | 0 | 0 | 0 | 0 | 1 | 0 | 0 | 0 | 0 | 0 |
| Consensus324 | 0 | 0 | 0 | 0 | 0 | 0 | 0 | 0 | 0 | 0 | 0 | 0 | 0 | 0 | 0 | 0 | 0 | 0 | 0 | 0 |
| Consensus325 | 0 | 9 | 0 | 5 | 3 | 3 | 3 | 0 | 1 | 1 | 0 | 0 | 12 | 0 | 4 | 44 | 2 | 38 | 1 | 1 |
| Consensus326 | 0 | 0 | 0 | 0 | 0 | 0 | 0 | 0 | 0 | 0 | 1 | 0 | 3 | 1 | 3 | 3 | 0 | 1 | 1 | 1 |
| Consensus327 | 0 | 1 | 0 | 0 | 0 | 0 | 0 | 0 | 0 | 0 | 0 | 0 | 0 | 0 | 0 | 0 | 0 | 0 | 0 | 0 |
| Consensus330 | 0 | 0 | 0 | 0 | 0 | 0 | 0 | 0 | 0 | 0 | 0 | 0 | 0 | 2 | 0 | 0 | 0 | 0 | 0 | 6 |
| Consensus331 | 0 | 0 | 0 | 0 | 0 | 0 | 0 | 0 | 0 | 0 | 0 | 0 | 0 | 0 | 0 | 0 | 0 | 0 | 0 | 0 |
| Consensus333 | 0 | 0 | 0 | 0 | 0 | 0 | 0 | 0 | 0 | 0 | 0 | 0 | 0 | 0 | 1 | 0 | 0 | 0 | 0 | 1 |
| Consensus334 | 0 | 1 | 0 | 0 | 0 | 0 | 0 | 0 | 0 | 0 | 0 | 0 | 1 | 1 | 0 | 0 | 46 | 0 | 0 | 1 |
| Consensus339 | 0 | 0 | 0 | 0 | 0 | 0 | 0 | 0 | 0 | 0 | 0 | 0 | 0 | 0 | 0 | 0 | 0 | 0 | 0 | 0 |
| Consensus341 | 0 | 0 | 0 | 0 | 0 | 0 | 0 | 0 | 0 | 0 | 0 | 0 | 14 | 1 | 5 | 2 | 0 | 5 | 0 | 1 |
| Consensus345 | 0 | 0 | 0 | 0 | 0 | 0 | 0 | 0 | 0 | 0 | 0 | 0 | 0 | 0 | 0 | 0 | 0 | 0 | 0 | 0 |
| Consensus348 | 0 | 0 | 0 | 0 | 0 | 0 | 10 | 0 | 0 | 2 | 0 | 9 | 9 | 11 | 29 | 30 | 5 | 63 | 14 | 523 |
| Consensus349 | 1 | 1 | 0 | 0 | 0 | 0 | 0 | 0 | 0 | 0 | 0 | 0 | 8 | 1 | 2 | 659 | 0 | 25 | 0 | 1 |
| Consensus351 | 0 | 0 | 0 | 0 | 0 | 0 | 0 | 0 | 0 | 0 | 0 | 0 | 0 | 0 | 0 | 0 | 0 | 0 | 0 | 0 |
| Consensus353 | 0 | 0 | 0 | 0 | 0 | 0 | 0 | 0 | 0 | 0 | 0 | 0 | 7 | 60 | 4 | 2 | 0 | 0 | 0 | 1 |
| Consensus355 | 0 | 1 | 0 | 0 | 0 | 0 | 0 | 0 | 0 | 0 | 0 | 0 | 0 | 0 | 0 | 0 | 0 | 0 | 0 | 0 |
| Consensus357 | 0 | 0 | 0 | 0 | 0 | 15 | 1 | 0 | 4 | 161 | 18 | 845 | 0 | 3 | 19 | 5 | 21 | 0 | 0 | 6 |
| Consensus359 | 0 | 0 | 1 | 0 | 0 | 0 | 0 | 0 | 0 | 0 | 0 | 0 | 0 | 0 | 0 | 0 | 0 | 0 | 0 | 0 |
| Consensus362 | 0 | 0 | 0 | 0 | 0 | 0 | 0 | 0 | 0 | 0 | 0 | 0 | 1 | 0 | 0 | 0 | 0 | 0 | 0 | 0 |
| Consensus369 | 0 | 0 | 0 | 0 | 0 | 0 | 0 | 0 | 0 | 0 | 0 | 0 | 0 | 4 | 1 | 0 | 0 | 0 | 0 | 0 |
| Consensus370 | 0 | 0 | 0 | 0 | 0 | 0 | 0 | 0 | 0 | 0 | 0 | 0 | 1 | 0 | 84 | 108 | 0 | 108 | 7 | 2 |
| Consensus374 | 0 | 0 | 0 | 0 | 0 | 0 | 0 | 0 | 0 | 0 | 0 | 0 | 0 | 0 | 0 | 0 | 0 | 0 | 0 | 0 |
| Consensus378 | 0 | 0 | 0 | 0 | 0 | 0 | 0 | 0 | 1 | 0 | 0 | 0 | 232 | 263 | 17 | 30 | 32 | 308 | 47 | 48 |
| Consensus379 | 0 | 0 | 0 | 0 | 0 | 0 | 1 | 0 | 0 | 0 | 0 | 0 | 3 | 0 | 0 | 0 | 0 | 0 | 0 | 0 |
| Consensus384 | 0 | 0 | 0 | 0 | 0 | 0 | 0 | 0 | 0 | 0 | 0 | 0 | 0 | 0 | 0 | 1 | 0 | 0 | 0 | 8 |
| Consensus388 | 0 | 0 | 1 | 6 | 1 | 0 | 0 | 0 | 0 | 0 | 0 | 0 | 0 | 0 | 0 | 0 | 0 | 0 | 0 | 0 |
| Consensus391 | 0 | 0 | 0 | 0 | 0 | 0 | 0 | 0 | 0 | 0 | 0 | 0 | 2 | 0 | 0 | 0 | 1 | 0 | 0 | 2 |
| Consensus398 | 0 | 0 | 0 | 0 | 0 | 0 | 0 | 0 | 0 | 0 | 0 | 0 | 0 | 0 | 0 | 0 | 0 | 0 | 0 | 0 |
| Consensus400 | 0 | 0 | 0 | 0 | 0 | 0 | 0 | 0 | 0 | 0 | 0 | 0 | 0 | 0 | 0 | 0 | 0 | 0 | 0 | 0 |
| Consensus405 | 0 | 0 | 0 | 0 | 0 | 0 | 0 | 0 | 0 | 0 | 0 | 0 | 0 | 0 | 0 | 2 | 0 | 0 | 0 | 0 |
| Consensus406 | 0 | 0 | 0 | 0 | 0 | 0 | 0 | 0 | 0 | 0 | 0 | 0 | 0 | 0 | 1 | 0 | 0 | 0 | 0 | 0 |
| Consensus407 | 0 | 0 | 0 | 0 | 0 | 0 | 1 | 0 | 0 | 0 | 0 | 0 | 0 | 0 | 0 | 0 | 0 | 0 | 0 | 0 |
| Consensus410 | 0 | 0 | 0 | 0 | 0 | 0 | 0 | 0 | 0 | 0 | 0 | 0 | 0 | 0 | 0 | 0 | 0 | 0 | 1 | 0 |
| Consensus417 | 0 | 0 | 0 | 0 | 0 | 0 | 0 | 0 | 0 | 0 | 0 | 0 | 0 | 0 | 0 | 2 | 0 | 1 | 0 | 2 |
| Consensus418 | 0 | 0 | 0 | 0 | 0 | 0 | 0 | 0 | 0 | 0 | 0 | 0 | 0 | 0 | 0 | 0 | 0 | 0 | 0 | 0 |
| Consensus419 | 0 | 0 | 0 | 0 | 0 | 0 | 0 | 0 | 0 | 0 | 0 | 0 | 0 | 0 | 0 | 0 | 0 | 0 | 0 | 0 |
| Consensus420 | 0 | 0 | 0 | 1 | 0 | 0 | 0 | 0 | 0 | 0 | 0 | 0 | 0 | 0 | 0 | 0 | 0 | 0 | 0 | 0 |
| Consensus421 | 0 | 0 | 0 | 0 | 0 | 0 | 0 | 0 | 0 | 0 | 0 | 0 | 0 | 0 | 4 | 0 | 0 | 0 | 0 | 0 |
| Consensus425 | 0 | 0 | 0 | 0 | 0 | 0 | 0 | 0 | 0 | 0 | 0 | 0 | 0 | 0 | 2 | 0 | 0 | 0 | 0 | 0 |
| Consensus426 | 0 | 0 | 0 | 0 | 0 | 0 | 0 | 0 | 0 | 0 | 0 | 0 | 0 | 0 | 0 | 0 | 0 | 0 | 0 | 0 |
| Consensus427 | 0 | 0 | 0 | 0 | 0 | 0 | 0 | 0 | 0 | 0 | 0 | 0 | 0 | 0 | 0 | 0 | 0 | 0 | 0 | 0 |
| Consensus428 | 0 | 0 | 0 | 0 | 0 | 0 | 0 | 0 | 0 | 0 | 0 | 0 | 0 | 1 | 0 | 0 | 0 | 0 | 0 | 0 |
| Consensus432 | 0 | 0 | 0 | 1 | 0 | 2 | 0 | 0 | 13 | 0 | 0 | 0 | 15 | 0 | 0 | 1 | 0 | 0 | 0 | 0 |
| Consensus435 | 0 | 0 | 0 | 0 | 0 | 0 | 0 | 0 | 0 | 0 | 0 | 0 | 0 | 0 | 0 | 7 | 2 | 0 | 0 | 0 |
| Consensus436 | 0 | 0 | 0 | 0 | 0 | 0 | 0 | 0 | 1 | 0 | 0 | 0 | 0 | 0 | 25 | 3 | 0 | 0 | 0 | 0 |

**Supplemental Data. OUTs generated based on sequencing reads of Experiment 2. (A )** Number of OUT reads in Experiment 2. **(B)** Number of OUT reads in Experiment 1.

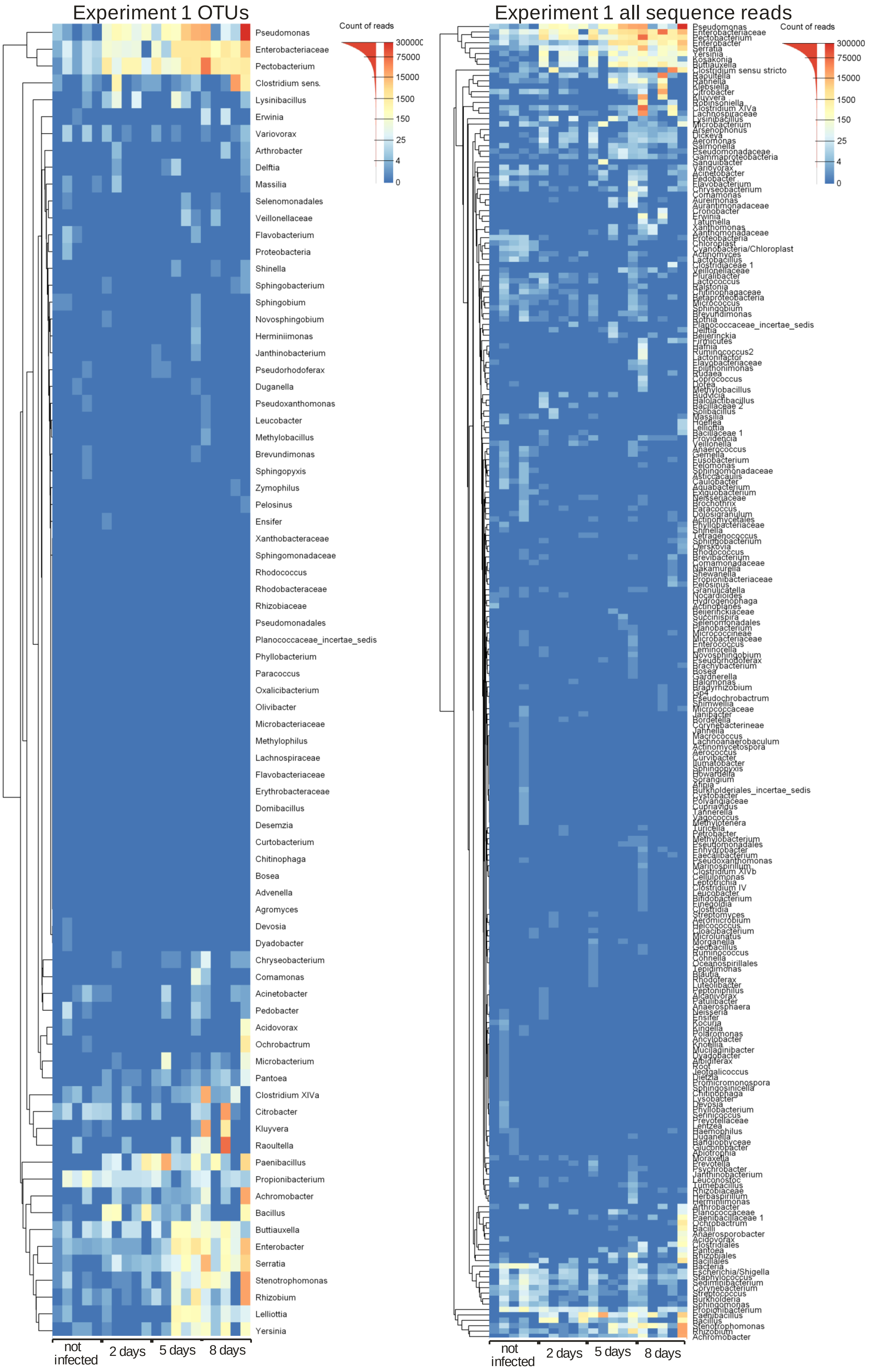


**Supplemental Figure S1. Heatmaps of Experiment 1**. (**A)** OTU sequences from Experiment 2 were used to cluster all similar sequences from Experiment 1. (**B**) Counted sequences reads of Experiment 1 classified by the RDP *multiclassifier* were normalized using inverse hyperbolic sine transformation. Heatmaps were made in R using pheatmap (parameters: clustering_method = average, clustering_distance = euclidean).

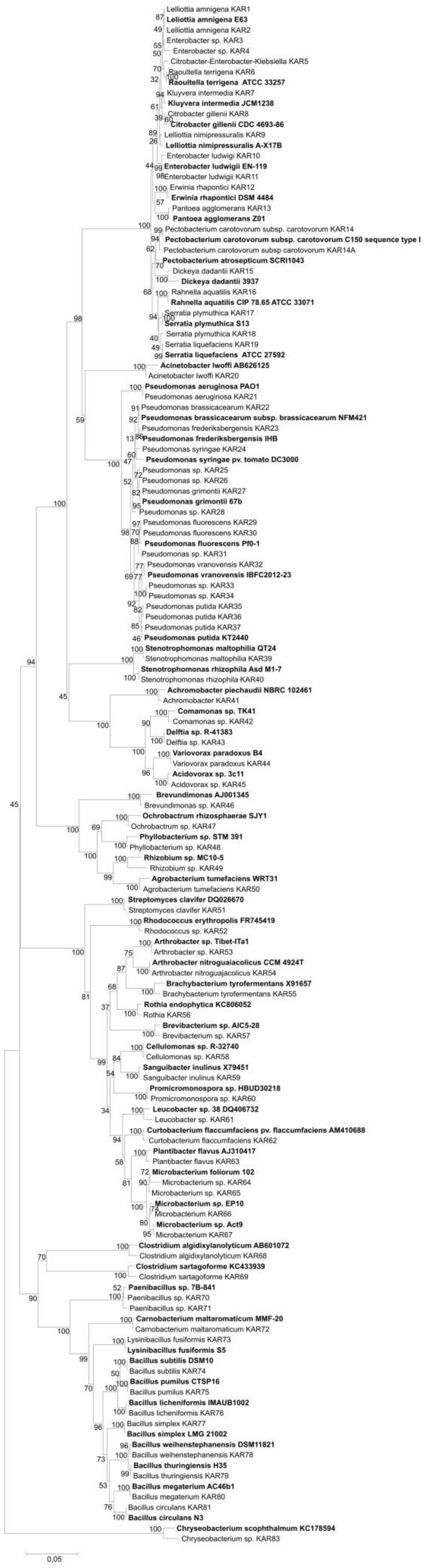

**Supplemental Figure S2. Phylogenetic tree of cultivable bacteria based on the 16S rRNA gene sequence comparison.** The tree was constructed based on the nearly full length 16S rRNA genes of cultivable bacteria and their nearest sequences (bold), as retrieved from the GenBank database.
